# Infant Formula Affordability Negatively Impacts Parental Wellbeing, Financial Security and Safe Feeding Practices in the UK

**DOI:** 10.1111/mcn.70228

**Published:** 2026-07-31

**Authors:** Amy Brown, Charlotte Walker, Aimee Grant, Sally Etheridge, Sabine Goodwin, Catrin Griffiths, Sara Jones, Holly Morse, Rhiannon Phillips, Rosalind Sharpe, Victoria Thomas, Victoria Sibson

**Affiliations:** ^1^ Faculty of Medicine, Health and Life Science Swansea University Swansea UK; ^2^ Centre for Lactation Infant Feeding and Translation (LIFT) Swansea UK; ^3^ Leicester Mammas Leicester UK; ^4^ Independent Food Aid Network London UK; ^5^ Cardiff Metropolitan University Cardiff UK; ^6^ University of Hertfordshire Hatfield UK; ^7^ Newcastle Upon Tyne Hospitals NHS Foundation Trust Newcastle UK; ^8^ First Steps Nutrition Trust The Food Exchange London UK

**Keywords:** advertising, conflicts of interest, food banks, food insecurity, infant feeding, infant formula, poverty, unsafe feeding practices, wellbeing

## Abstract

In the UK, media reporting has highlighted the impact of high infant formula prices upon parent wellbeing and feeding practices. We explored this issue through surveys (*n* = 261) and interviews (*n* = 20) with parents who were struggling to afford infant formula, and surveys (*n* = 137) and interviews (*n* = 21) with stakeholders in infant feeding, parenting and food insecurity. Almost all parents and stakeholders identified a detrimental impact upon parental mental and physical health, relationships, social and emotional wellbeing. Parents described feeling shame, anxiety and sadness, compounded if mothers wanted to breastfeed but could not. Some continued to breastfeed through pain whilst others stopped because they worried their diet was insufficient. To afford milk some parents reported restricting their own food or heating, returning to work early and getting into debt. Almost half sourced formula, including opened products, from social media marketplaces (46.0%) or bought formulas marketed for older babies (45.2%). Over a third (39.1%) reported using an unsafe feeding practice, including using leftover formula for a feed later (28.7%), reducing sterilisation (21.5%), and watering down feeds (13.8%). Many realised these were not recommended practices and felt guilty and anxious. Although a third were supported by Healthy/Best Start allowance, many families did not qualify for benefits or financial support and often had two working parents. Reduced maternity pay, high childcare costs and the broader cost‐of‐living crisis, affected affordability. Our data highlight an urgent need for intervention, including enhanced emergency provision pathways, stronger controls on formula marketing, infant formula price regulation, and enhanced breastfeeding support.

## Key Messages

- High infant formula costs are a social justice issue, placing a moral burden on parents through impacts on infant health, family wellbeing and financial security
- High prices led parents into debt, unsafe feeding practices, sourcing products on social media marketplaces, going without food themselves or stealing formula
- Mothers who had experienced breastfeeding difficulties felt doubly let down at a lack of breastfeeding support and high formula prices showing a need for investment across infant feeding
- There is an urgent need for government regulation of infant formula marketing and price increases alongside enhanced emergency feeding pathways and access to financial support

## 1 Background

The recent cost‐of‐living crisis saw rapid increases in food prices in many countries from 2017 onwards (Braun 2022). In the UK inflation on food and non‐alcoholic drinks rose sharply, peaking at 19.2% in 2023 (Office for National Statistics 2024). However, infant formula, which was already expensive, exceeded this, rising by an average of 24%, with increases up to 45% between March 2021 and April 2023 (First Steps Nutrition Trust 2023). The recent Competition and Market Authority's (CMA) infant formula and follow‐on formula market report identified that this increase was greater than increases in production costs, with manufacturers variable gross margins between 50% and 75% in 2024 and retail gross profit margins between 18% and 22% (Competition and Markets Authority 2025). This is a significant issue because infant formula is not a discretionary product. When infants are not breastfed or not fully breastfed, infant formula is needed to support growth and development (NHS 2023).

Over half of babies in the UK receive formula during their first week, with 88% receiving some formula milk by 6 months of age (McAndrew et al. 2012). Although breastfeeding rates differ between countries, partial and exclusive formula feeding in the first year of life is common in many countries, particularly in Western regions (Victora et al. 2016). Globally, fewer than half of infants are exclusively breastfed for the first 6 months of life (UNICEF 2025).

Although some mothers decide to or need to use infant formula, many set out planning to breastfeed, but complex factors work against this. A lack of investment in skilled breastfeeding support, poor social and cultural understanding of how breastfeeding works, isolation of new families, a lack of workplace legislation and predatory formula marketing all contribute to a formula dominant culture means that many mothers stop breastfeeding before they are ready (Pérez‐Escamilla et al. 2023; Brown 2017). Parents on a lower income are disproportionately affected by this as they are more likely to use formula milk (Quigley et al. 2023), especially when breastfeeding support is scarce (Fitzsimons & Vera‐Hernández 2022). The health impacts of not being breastfed are also greater in more deprived households (Quigley et al. 2006).

Articles in the UK media have highlighted the detrimental impact of rising costs of formula milk upon parents' wellbeing with examples of unsafe feeding practices such as watering down feeds (BBC News 2023; The Guardian 2023). These experiences are echoed in reports from charities, third sector organisations and parliamentary groups (FEED UK 2022; British Pregnancy Advisory Service 2023; All‐Party Parliamentary Group on Infant Feeding and Inequalities 2018). This is reflected in limited research with food insecure mothers from other countries. A study in Canada found that some used powdered cow's milk or watered‐down formula to make it last (Frank 2015), whilst another study in North Carolina reported use of cow's milk and early introduction of solids (Hardison‐Moody et al. 2018). These practices may have a detrimental effect on younger babies' growth, gut health and risks of iron deficiency anaemia (Scientific Advisory Committee on Nutrition 2018).

Broader literature has explored the impacts of infant feeding experiences upon mental health. Breastfeeding difficulties or having to stop sooner than planned can have a significant negative impact upon maternal mental health (Brown 2018; Tanganhito et al. 2020). Mothers who use infant formula often describe feelings of shame, judgement and guilt (Jackson et al. 2021), whilst many parents regardless of feeding method, feel unsupported (Grant et al. 2022; Wheeler et al. 2024). However, although research has explored the reasons and motivations for using infant formula particularly amongst lower income groups (Cook et al. 2021; Grant et al. 2019), and the broader impacts of food poverty upon health (Myers 2020; Ejiohuo et al. 2024), no data has been published from a UK perspective on the experience of struggling to afford to purchase infant formula.

Understanding the impact that infant formula affordability, especially in light of recent further price increases, has had upon parental well‐being and infant feeding practices is vital in developing strategies to support families. The aim of this study was therefore to explore the experiences and perspectives of parents, professionals and volunteers working in infant feeding, parenting and poverty support to understand the breadth and scale of the impact of prices upon parental well‐being, affordability strategies and feeding practices.

## 2 Methods

### 2.1 Design

Mixed‐methods study using cross‐sectional online surveys and interviews with parents and professionals/volunteers working in infant feeding, parenting and poverty support.

### 2.2 Participants

This data forms part of a larger mixed‐methods study of parents and professionals/volunteers in the UK exploring their views and experiences of the impact of high infant formula milk prices upon families. The wider study included the views of 642 UK parents with a baby aged 0–2 months (281 parents who struggled to afford infant formula and 361 who did not). Additionally, 158 professionals and volunteers working in infant feeding, parenting and food poverty participated. Both parents and professionals/volunteers were asked about their views on different options (such as increases to financial benefits, capped prices and no‐cost infant formula via a health professional or food bank) to support families who could not afford infant formula.

This data here draws on the additional experiences of a subset of parents who were struggling to afford infant formula for their baby. These parents answered an additional set of questions around how not being able to afford infant formula made them feel and how they managed to secure infant formula milk for their baby.

Parent inclusion criteria for this study were: UK‐based, aged 16+ , baby aged 0–12 months old, partial or exclusive formula feeding and experiencing significant financial difficulties that impacted their ability to purchase infant formula for their baby. We measured financial difficulties by asking parents if they agreed with the following description ‘*Have you ever found yourself struggling to afford to buy formula milk but managed to do so by making significant cuts elsewhere such as going without food yourself, not being able to heat your home, not paying a bill, taking on extra work or getting into debt?* Or *you've had to ask for help (from family, friends, a health professional, food bank or charity) so your baby doesn't go without?’*.

Parents who agreed with the statement completed the items on how it made them feel and how they accessed infant formula. Parents who did not agree did not see these questions in the survey. We chose to use this definition rather than income level because we hypothesised that families may have experience of struggling to afford formula due to increased essential costs such as housing, utilities, food and childcare despite not having the lowest incomes and thus not being eligible for financial assistance.

Additionally, professionals or volunteers working in infant feeding, parenting support or food insecurity support took part. Inclusion criteria were: UK‐based, aged 18+ , working directly with parents or in a management/leadership role, and a core part of the role relating to infant nutrition, parent wellbeing or food poverty. We refer to these individuals as ‘stakeholders’. Exclusion criteria for both parents and professionals/volunteers included not being able to give informed consent, or not able to complete the survey or interview in the English language.

Full ethical permission for the study was gained from Swansea University School of Health and Social Care Research Ethics Committee. Participants gave informed consent, and all aspects of the study were carried out in line with the Declaration of Helsinki 2024 (World Medical Association 2025).

### 2.3 Ethics Statement

Ethical approval was granted by the Swansea University School of Health and Social Care Research Ethics Committee.

### 2.4 Measures

Participants completed online questionnaires containing both closed and open questions, hosted by Qualtrics. Questions were developed based on themes that have been recently presented in parents' experiences in the UK media, in charity reports and research outside the UK as reported in the background section. We did not seek to measure experiences representatively across the whole parent population who use formula milk (i.e. to estimate how many are struggling to do so), but instead specifically explore the experiences of those who were struggling to afford to feed their baby.

Both the parent (Appendix one) and stakeholder (Appendix two) surveys were specially designed by the research team for this project. Themes were based on existing relevant research literature and reports (FEED UK 2022; British Pregnancy Advisory Service 2023; All‐Party Parliamentary Group on Infant Feeding and Inequalities 2018; Frank 2015; Hardison‐Moody et al. 2018) and media articles discussing high formula prices and the impact upon parents (BBC News 2023; The Guardian 2023). Survey questions were tested across our team and then piloted via two of our PPI reps (parents who had experience of struggling to afford infant formula) and stakeholders in our team who worked in parenting, infant feeding and food crisis support to check for clarity. Minor changes were made for readability with reviewers agreeing questions were suitable, sensitively written and reflected their experiences.

For the parent survey, items that are relevant to this study included demographic questions for the parent and baby, items to capture how babies were fed, and how parents afford/access infant formula, such as borrowing money, receiving it as a gift or managing to source it from a health professional scheme or similar. We then identified a set of potentially ‘unsafe feeding practices’ that parents might be resorting to, in order to access infant formula or make it stretch further. This included aspects such as using leftover formula for a later feed, watering down milk, reducing sterilisation, using cows' milk or milk marketed at older babies, or sourcing milk from social media marketplaces. Finally, we developed a set of questions to measure the impact of not being able to afford infant formula on parent wellbeing. Items included aspects such as mental health, physical health and relationships with others and again came from descriptions given in similar research of media articles.

Alongside the indicator described above of financial difficulties, parents indicated whether they received the Healthy/Best Start allowance. This scheme is available to parents who are pregnant or have a child under 4 years old with an earned income £408 or less from employment each month. For parents with a baby 0–12 months old, it was worth £8.50 per week (at the time of data collection). It can be spent on infant formula but also fruit, vegetables cows' milk and pulses. We used this as an additional indicator of low income, but it can only serve as an approximation as many parents are unaware of it, with some ineligible due to no right to access public funds. It is also a good indicator to illustrate how parents who fall outside the qualifying income criteria can still be experiencing significant financial difficulty due to high cost‐of‐living expenditure on basics and essentials. Additionally, parents were asked whether they accessed support via food and baby banks. Food banks in the UK provide emergency food and practical support to people who have been left without enough money to live on. Baby banks provide essential items for babies and children to families in need.

For the stakeholder survey (Appendix two), themes mirrored those found in the parent survey but asked stakeholders to reflect on whether they saw these behaviours and impacts in parents they supported or had heard about it in their role.

Interview questions (for both parents and stakeholders) reflected themes explored in the survey to allow participants to talk about them in more detail. Interviews were semi‐structured, allowing for prompts and exploration of new ideas that arose. Again, interview questions reflected the themes of the main larger study, but specific items were relevant to this study. These can be found in Appendix three but for parents included questions such as ‘How easy or difficult is it for you to buy the formula your baby needs?’, ‘How does this make you feel?’ and ‘Have you ever had to cut corners or do things you thought might not want to because you couldn't afford enough formula?’. For stakeholders questions explored similar themes but through the lens of the impact they saw high infant formula prices had upon families.

Please note, when referring to different types of milk parents were giving their baby we use ‘infant formula’ to refer to milk suitable from birth to 12 months. Sometimes referred to as ‘first stage infant formula’ these milks are the only products recommended for purchase by the (NHS 2023). We use the term ‘follow on’ milks for those marketed for babies 6–12 months and ‘toddler milks’ for those marketed for babies 12 months and older. Neither of these are recommended products. When we consider the use of differing types of formula products given, we use the term ‘formula milk’.

### 2.5 Procedure

Data were collected between March and August 2025. First, adverts for the survey were shared on social media, with one advert for parents and one for professionals/volunteers. The survey had the option to leave contact details (stored separately) as an expression of interest to take part in an interview. However, additional adverts (again one for parents and one for professionals/volunteers) were shared to advertise the interview study asking for expressions of interest to take part. In all cases, posts included encouragement for other individuals and organisations to share. Adverts contained information about the study, data collection and inclusion criteria. Details of incentives for parents were given, including the option to be added to a prize draw to win one of 5 x £20 shopping vouchers for the survey (details stored separately) and a £20 shopping voucher for taking part in the interview.

Posts were shared on the academic/organisational social media pages of the diverse study team (academic, infant feeding, child health, parenting and food bank organisations). This included Facebook, Instagram, LinkedIn and X. The study team members who were from organisations also shared study adverts on their web pages, with mailing lists and in face‐to‐face support groups they delivered. The study team have a wide social media reach and with sharing of posts sufficient participant numbers and diversity of participants were reached without needing paid posts. Hashtags such as #feedingbabies #costofliving #parenting #money were used to distribute the survey wider that connected accounts.

Study adverts for the surveys contained a link to the study information sheet. For the survey, consent questions were loaded, followed by the survey if the participant consented. Once completed a debrief statement was given, including contact details for support organisations. Paper copies of the survey could be made available, but no participant requested this.

For the interviews, the study adverts again contained a link to the study information sheet. If potential participants wanted to be considered for interview, they answered some brief demographic/work role questions and left preferred contact details (email or phone number). A purposive sample of participants was then invited to interview. For the parents, selection prioritised ensuring a range of ages, ethnic groups and partial versus exclusive formula feeding. For the professionals/volunteers, potential participants were split into core roles (e.g. infant feeding advisor, parenting support worker, food bank staff, charities) and a selection was invited to interview, considering a mix of local and national roles.

Invitation to interview included sending the study information sheet again, consent questions and option to confirm they would like to take part. An interview was then arranged if they wished to proceed. A reminder was sent if no response was received within 7 days. Interviews were conducted and recorded via MS Teams or Zoom, with one interview conducted over the telephone due to technical difficulties. To support parents who might not be able to afford digital access, a prepaid SIM card was an option. Face‐to‐face interviews were advertised but all participants preferred an online format, often due to childcare and accessibility.

At the start of the interview participants were reminded of the study aims and procedures and asked to reconfirm consent, noting that they could stop at any time and did not have to answer any questions they did not want to. Participants could turn their camera off during the recording, after initial introductions (which were not recorded). Participants could discuss topics in whichever way was most comfortable, aiming to cover the areas in the topic guide. At the end of the interviews, participants were debriefed and given information about support organisations. They received a £20 shopping voucher for their time.

### 2.6 Data Analysis

For the survey data, quantitative data were analysed using SPSS version 29. Descriptive analysis was carried out on the closed‐response survey data to provide frequencies of response categories. Non‐parametric Chi‐square tests were used to identify associations between categorical data provided on infant feeding practices, well‐being and demographics. T tests and Pearson's correlations were used to explore associations between demographic background and parental wellbeing. To compare differences in experience for those eligible for financial support based on low income, chi‐square and MANOVA were used to compute frequencies of behaviour/degree of impact between those accessing the Healthy/Best Start scheme and not.

Interviews were transcribed and a simple descriptive approach (Sandelowski 2010). This analysis approach focuses on providing a straightforward, comprehensive summary of experiences and behaviours without deep interpretation, theory development, or heavy manipulation of data. It is often used to understand a topic from a participants' perspective and to describe current events rather than developing theory (Villamin et al. 2025). A thematic analysis combining both interview and open‐ended survey questions was conducted. NVIVO was used for analysis. A deductive coding approach was followed to explore brand decisions, affordability strategies, parental wellbeing, and feeding practices. Further inductive codes were generated from the data for additional themes that had not been hypothesised to emerge.

To minimise bias, principles of trustworthiness were followed, considering the criteria of credibility, transferability, dependability and confirmability (Lincoln and Guba 1985). To enhance credibility, one author (CW) immersed themselves in the data, reading through responses from each participant and across questions for all participants and re‐reading them iteratively to develop themes. To enhance reliability of the analysis, another author (AB) read all transcripts and reviewed proposed themes and subthemes. Where disagreement occurred, themes were discussed until agreed. To increase triangulation, a convergent narrative integration approach (Creswell and Plano Clark 2018) was used to present findings from the open‐ended and closed‐item survey data and interviews in line with the research questions rather than considering quantitative and qualitative data in separate sections. Each participant survey and interview response was treated as a data set and scanned for responses across multiple open‐ended boxes and interview questions to integrate themes together.

Examples of participant quotes are provided for each theme and subtheme, ensuring we drew on the experiences of multiple participants to enhance transferability. Detailed notes and iterations of drafts of findings were kept and discussed across the research team to increase dependability. Finally, confirmability was enhanced by the reflexivity of the study team in terms of their own parenting and professional experiences. We also shared the findings with PPI group members (parents with experience of struggling to afford infant formula), to ask if our findings and interpretation reflected further lived experiences. We sent summaries of the research to participants who wanted to receive them, allowing for feedback before we finalised our reporting.

## 3 Results

Two hundred and sixty‐one parents fully completed the survey (258 mothers and 3 fathers). Forty‐six participants had partial data and were excluded due to not completing sections of data relevant to analysis. Most in this category completed < 40%, leaving the survey after initial questions were answered (rather than skipping through to the end). The majority of participants in our study were mothers (98.6%) but participation was open to and included fathers and therefore we refer to ‘parents’ throughout, apart from where data was only generated by participants who were mothers. Further demographic data can be found in Table 1.

**Table 1:** Parent demographic background for survey participants.

Category	Sub‐category	*N*	%
Parent	Mother	258	98.9
	Father	3	1.1
Age	18–24	74	28.4
	25–29	71	27.2
	30–34	72	27.6
	35–39	36	13.8
	40+	8	3.0
Education	No formal qualifications	9	3.4
	GCSE or equivalent	53	20.3
	A level or equivalent	129	49.4
	Degree or equivalent	54	20.7
	Postgraduate qualification or equivalent	16	6.1
Ethnicity	Asian or Asian British: Bangladeshi	9	3.4
	Asian or Asian British: Chinese	4	1.5
	Asian or Asian British: Indian	20	7.7
	Asian or Asian British: Pakistani	19	7.3
	Any other Asian background	1	0.3
	Black or Black British	21	8.0
	Mixed or Multiple	22	8.4
	White British or Irish	148	56.7
	White (other)	16	6.1
	Gypsy or Irish Traveller	1	0.3
Number of children	One child	126	48.2
	More than one child	135	51.7
Country	England	158	60.5
	Northern Ireland	20	7.6
	Scotland	18	6.9
	Wales	65	24.9
Disability or chronic health issue	Yes	58	22.2
	No	203	77.8
Neurodivergence	Yes (including self‐identified)	71	27.2
	Not sure	11	4.2
	No	179	68.5
Work status	Yes full time	39	14.9
	Yes part time	69	26.4
	Yes zero hours	4	1.5
	Maternity/paternity leave	108	41.4
	No	36	13.7
Live with a partner	Yes	195	74.7
	No	66	25.3
Partner in work (*n* = 195)	Yes full time	108	55.3
	Yes part time	32	29.6
	Yes zero hours	11	5.6
	Maternity/paternity leave	4	2.0
	No	40	20.5

For babies, 176 parents responded in relation to having a baby aged 0–5 months (67.4%) and 85 (32.7%) had a baby 6–12 months. All babies were formula fed, 79 (30.3%) had breast milk, 146 (55.9%) solid foods, and 30 (11.5%) had cow's milk as a drink. We refer to those who were receiving formula milk alongside breastmilk as being mixed fed (e.g. 79, 30.3% of babies). For babies aged 6 months and younger (before the age solid foods are recommended), 66/176 babies (37.5%) received solid foods and 15 (4.1%) cow's milk as a drink.

One hundred and thirty‐seven stakeholders took part in the survey with only 10 responses not included due to partial completion. Participants included 83 (60.6%) health or social care professionals, 30 (21.9%) paid peer workers, 39 (28.5%) volunteers and 14 (10.2%) strategic, policy or managerial roles. Overall, 122 (89.1%) described their role as including infant feeding support, 76 (55.5%) parenting support, and 31 (22.6%) food or baby bank support. Specific roles included midwives, health visitors, infant feeding leads, antenatal teachers, breastfeeding counsellors and peer supporters, family health workers, food bank volunteers, child development practitioners and parenting charities. One hundred and twenty‐eight (93.4%) worked face‐to‐face with parents, whilst 9 (6.6%) did not.

Twenty parents (19 mothers and 1 father) also took part in interviews. Thirty‐five parents who met inclusion criteria expressed interest in taking part. Table 2 shows the parent interview participant demographic background and pseudonyms. A further 21 stakeholders took part in interviews, including infant feeding specialists and peer workers, public health specialists, NHS staff, managers of infant feeding services, food bank staff and baby bank managers. Sixty‐two expressions of interest were received.

**Table 2:** Parent demographic background for interview participants (using pseudonyms).

Pseudonym	Age	Ethnicity	Baby age	Feeding method
Hedi	25– 34	White	12 weeks	Formula milk
Zoe	25– 34	White	11 days and 12 m	Formula milk
Rosie	25– 34	White	8 months	Formula milk
Olivia	25–34	White	7 months	Mixed feeding
Leila	25– 34	Asian	6 months	Formula milk
Abeni	—	Black/African/Caribbean	8 months	Formula milk
Holly	25– 34	White	4.5 months	Mixed feeding
Emma	25– 34	Black/African/Caribbean	8 months	Formula milk
Nicola	35– 44	White	11weeks	Formula milk
Lara	35– 44	White	5 months	Formula milk
Marie	25– 34	Black/African/Caribbean	12 months	Mixed feeding
Lisa	35– 44	White	19 weeks	Formula milk
Malik	35– 44	White	7 months	Formula milk
Katie	35– 44	White	4 months	Formula milk
Lesley	25– 34	White	4 months	Formula milk
Catrina	25– 34	Black/African/Caribbean	5 months	Mixed feeding
Alayah	—	Asian	9 months	Formula milk
Lucy	25– 34	Black/African/Caribbean	7 months	Formula milk
Aditi	—	Asian	7 months	Mixed feeding
Alice	25– 34	White	4 months	Formula milk

Findings are presented, integrating data from both surveys and interviews, under five key themes. Four of these themes were identified in study development: Cost of formula milk and brand decisions, strategies used to afford/access infant formula, impacts of infant formula prices upon parent wellbeing, and impacts upon infant feeding practices. A further fifth theme was identified: impact upon breastfeeding. For parent qualitative responses, survey participants are identified by demographic descriptors and interview participants are identified by their pseudonym. Stakeholders are described by a broad role to allow anonymity.

### 3.1 Cost of infant formula and strategies used to afford/access infant formula

Parents were asked how they paid for/accessed milk other than through wages/savings. A minority had accessed formula from food/baby banks (*n* = 12, 4.6%) and health professionals (*n* = 45, 17.2%). Although 94 (36.01%) were supported by Healthy/Best Start allowance, many did not qualify for this due to their income (or believed they would not) or not having recourse to public funds due to status such as a Refugee, Asylum seeker, or a temporary migrant.

Other methods included 157 (60.2%) had received it as a gift from friends and family, 99 (37.9%) had it given to them by ‘someone they didn't know’ via social media (compared to 46% who ever sourced formula via social media), 120 (46.0%) used credit cards, 37 (14.2%) had got free samples from companies, 26 (10.0%) had taken it from a shop without paying. Parents also reported a series of coping strategies to afford formula, such as not heating their homes, skipping meals or returning to work early (Table 3). Some of these represented smaller actions in order to afford the additional costs of formula (i.e. borrowing money), whilst others were indicative of wider financial issues affecting the family, which made formula more difficult to afford (i.e. selling/stopping using a car). There were no significant associations between accessing Healthy/Best Start or not and strategies used.

These behaviours were prevalent throughout the interviews and open‐ended survey boxes. Increased debt (‘*we are now in credit card debt and have missed bill payments*’, skipping meals (‘*my husband was even skipping meals’*) and taking on further work (‘*my partner has taken on another job and barely sleeps. He's a broken man’*), including work women were unhappy with (‘*I've taken online and call jobs that I wouldn't usually do including trying sites like onlyfans’.) were common*. Stakeholders also saw these experiences, with many witnessing debt (‘*Parents go into debt with household bills’.*) or parents prioritising feeding their baby over all else *(‘People tend to put the baby first and go without themselves’)*.

### 3.2 Impacts of infant formula prices upon parent wellbeing

Parents, and stakeholders who worked directly with families (*n* = 123), were asked how formula costs affected parental wellbeing. Almost all participants felt that it affected parents' mental, physical and social wellbeing, with 89.3% of parents agreeing it negatively affected how they felt about themselves as a new parent, 86.6% their mental health, 80.8% their ability to relax and enjoy life and 73.9% their physical health (Table 4). A MANOVA found significant differences in impact for physical health [F (1259) = 5.862, *p* = 0.016] with those not accessing Healthy/Best Start more likely to state that they were affected.

**Table 3:** Strategies used to purchase formula milk.

	Full sample		Access healthy/Best Start		Do not access healthy/Best Start	
	N	%	N	%	N	%
Gone without basic new clothing for myself	166	63.6	59	62.8	107	64.1
Borrowed money that is difficult to pay back	123	47.1	46	48.9	77	46.1
Not paid bills on time	112	42.9	38	40.4	74	44.3
Sold important items	102	39.1	40	42.6	62	37.1
Not heated our home as much as we needed	97	37.2	41	43.6	56	33.5
Gone without necessary things, e.g. optician, prescriptions, dentist	81	31.0	34	36.2	47	28.1
Taken on more work (over and above typical full‐time hours)	80	30.7	25	26.6	55	32.9
Gone without meals myself	55	21.1	22	23.4	33	19.8
Gone back to work from maternity/paternity leave early	55	21.1	16	17.0	39	23.4
Sold/stopped using a car	34	13.0	9	9.6	25	15.0

**Table 4:** Impact of high infant formula milk costs upon parental wellbeing (showing proportion who strongly agree or agree).

I feel that not being able to afford formula milk has negatively affected my	Parents						Professionals/volunteers	
	Full sample		Access Healthy/Best Start		Do not access Healthy/Best Start		N	%
	N	%	N	%	N	%		
Feelings about being a parent	233	89.3	84	89.4	149	89.2	132	98.5
Mental health	227	86.6	80	85.1	146	87.4	132	97.8
Ability to relax and enjoy life	211	80.8	76	80.9	135	80.8	131	98.5
Ability to concentrate on work or other responsibilities	210	80.5	71	76.2	139	83.5	‐	‐
Time on maternity/paternity leave	206	78.9	75	79.8	131	78.4	‐	‐
Relationship with partner	201	77.0	69	73.4	132	79.0	117	88.6
Physical health	193	73.9	74	78.7	119	71.2	109	82.6
Relationship with baby or children	95	36.4	36	38.3	59	35.4	118	89.4

Certain demographic groups were more significantly affected. Those who were diagnosed or identified as neurodivergent were significantly more likely to report that it had affected their mental health compared to those who were neurotypical [*t* (259) = −2.159, *p* = 0.016] and physical health [*t* (259) = −3.055, *p* = 0.001]. Younger parents were significantly more likely to report stronger negative impacts upon their experience of maternity/paternity leave than older parents (*r* − 0.147, *p* < 0.017). Parents with a university level qualification were significantly more likely to feel that it had negatively affected their relationship with their baby than those without [*t* (259) = 2.55, *p* = 0.006]. No significant associations/differences were found for age, disability status or between ethnic groups.

Parents and stakeholders expanded on these impacts in the interviews and open‐ended boxes.

#### 3.2.1 Anxiety and Stress

Many parents described the significant anxiety, fear and stress they were experiencing.

‘It is upsetting and scary and it makes me feel very down and anxious. I don't always know that I'm going to have enough milk to last and worry about how to find that money a lot? I worry about prices going up even more, or if other bills go up or we get an unexpected one it will wipe us out’.

(Survey: mother, age 30–34, White, baby 6 months, exclusive formula)

These feelings were also observed by the stakeholders.

‘It increases stress levels and causes parents to spend a lot of time balancing finances. This reduces the time and ability of parents to be responsive and support the baby's wider development. They are also likely to reduce their own food intake to reduce wider costs to support the purchase of formula. This hunger and sub‐optimal nutrition will affect their own physical and mental health and their ability to cope with the stresses of parenting’.

(Survey: Infant feeding and parenting supporter)

#### 3.2.2 Sadness

Sadness, grief and loss were common themes across the interviews and surveys

‘I cry all the time now. I'm meant to be the one who looks after her and protects her and I'm failing her all because I couldn't get feeding right and now can't afford her milk properly’.

(Survey: mother, age 30–34, Black African, baby 3 months, mixed feeding)

Some felt that the experience had caused or exacerbated a diagnosis of postnatal depression

‘Heartbroken that such a wonderful time should be so hard. I've got postnatal depression and cost of everything and worrying about my baby is a big part of it’.

(Survey: mother, age 25–29, White, baby 4 months, mixed feeding)

#### 3.2.3 Shame and Embarrassment

Others felt shame and embarrassment that they needed support to provide for their baby

‘It's actually hurtful that it's difficult for me, as a parent, to be able to provide formula for my baby. And sometimes it really hurts my ego as a mum to not be able to provide much for my child’.

(Interview: Marie, Mother, aged 25–34, Black, baby 12 months, mixed feeding)

This could be particularly strong if they had to rely on others for help

‘At my age I shouldn't be asking, but it's only because I'm a student midwife I am asking and the lack of funds. Well, it's embarrassing, really. Isn't it shameful? Because, especially at my age, because, like, you think you have kids, you're the provider, aren't you? So yeah, it's just shameful, really’.

(Interview: Katie, mother, aged 35–44, White, baby 4 months, exclusive formula)

However, some felt such shame that they felt they couldn't ask others for help or admit they were struggling.

‘I pretend to family that we're using {expensive brand} as that's what everyone uses but we're not. I keep old tubs out on the counter and have even put {cheaper brand} formula in it. It sounds a bit crazy written down but I want people to think I can afford to buy my baby the milk I want’.

(Survey: mother, age 19–24, White, baby 3 months, exclusive formula)

This included hiding their experiences from health professionals

‘It's come this whole thing about feeling so ashamed to have a conversation with actually someone who is very trusted. You know, a health visitor, someone that comes into your home’.

(Interview: Catrina, Mother, 25–34, Black, baby 5 months, mixed feeding)

Professionals and volunteers often saw parents who felt this way

‘They feel ashamed and that they are not good enough parents’.

(Survey: community public health nurse)

#### 3.2.4 Feeling Let Down and Frustrated

Two strong emotions in both the interviews and survey were feeling let down and frustrated.

‘I cant get past the fact that formula is food for babies and theres so little help when you cant afford it’.

(Survey: mother, age 30–34, Bangladeshi, A level, exclusive formula)

Others were frustrated at the lack of action despite growing awareness of the needs of many parents.

‘I feel frustrated at the lack of action around this. There's lots of talk about it but nothing seems to be happening to help people like me’.

(Survey: mother, age 19–24, Pakistani, baby 4 months, exclusive formula)

This appeared to be particularly true for the many parents who were from working households. Many had asked for help such as the Healthy Start allowance but had been turned down due to income.

‘I mean, I was working as well, and I'm on maternity. But our bills are like quite high, anyway, and like our mortgage went up and things like that. It's like we both earn like an okay amount between us, but actually, we, you know, we're still like struggling with it. I feel like it's the people kind of like in the middle that are struggling the most, just because like, I suppose if you're on a lower income, you do get help, and you do get like vouchers and stuff. But we're like over the amount’.

(Interview: Rosie, mother, aged 25–34, White, baby 8 months, exclusive formula)

These feelings were echoed by the stakeholders

‘(they feel) Powerless. Failures. Let down’.

(Survey: Health visitor)

#### 3.2.5 Awareness and Anger at Manufacturers and Industry

Another central theme was the growing awareness and anger at how much profit was being made on infant formula milks. Parents were aware that infant formula was a necessary product for babies who were not breastfed, had heard that profit margins were high, and were angry about this.

‘It's not just the price of the milk is it? It's the price of everything. Everything has gone up and I won't scrimp on feeding my baby but we're getting more and more into debt for everything. Then you find out they make loads of profit from milks and its not right is it?’

(Survey: mother, age 25–29, White, baby 1 month, Mixed feeding)

These feelings were often particularly strong when mothers had wanted to breastfeed but had been unsuccessful and felt therefore that their formula use was not a choice.

‘It's crazy that people are kind of making so much money off of this. I'd always wanted to breastfeed. But then for people who trust me, blood, sweat, and tears tried to breastfeed and then wasn't able to. Then I [was] kind of stuck with this financial burden’.

(Interview: Olivia, mother, aged 25–34, White, baby aged 7 months, mixed feeding)

Stakeholders also shared frustration at the cost and profits of infant formula

‘(The price) should be capped, I think, if you're providing an essential food source for babies, I think there should be a maximum cap of what you can have because profits profit. They're all going to make their profit anyway. But it should be a reasonable profit, not at the family's expense. I understand that companies have to make a profit. I'm not naive, but you don't need to make 45% raises’.

(Interview: Baby bank founder)

#### 3.2.6 Impact on Relationships

Lots of parents described how the increased stress of not being able to afford infant formula was impacting on their relationships. Sometimes this was directly with their partner, causing arguments at home

‘We argue so much more because of money now. My husband is proud and upset that he can't look after his family and that I have to work to be able to afford the normal stuff in life. It kills him when we run out and are getting more and more into debt to buy stuff we need’.

(Survey: mother, age 19−24, White Irish, baby three months, Mixed feeding)

Others barely saw their partner because they had taken on increased work, or they were so tired after long working hours that there was no energy left for each other

‘We don't have much time together any more as partner is always working’.

(Survey: mother, age 19–24, Indian, baby 5 months, exclusive formula)

It could also cause a lot of tension with family when support was not offered, or jealousy when others did not struggle in the same way.

‘I feel jealous of friends who don't have the same struggles I do and take things for granted like knowing they can feed their baby’.

(Survey: mother, age 25–29, White British, baby 3 months, exclusive formula)

Stakeholders often saw this play out

‘A failure. That they can't provide for their baby's basic needs. This could lead to issues between parents relationships. They may isolate themselves as they don't want to be seen to be struggling’.

(Survey: Specialist Midwife for Infant Feeding)

#### 3.2.7 Experience of Being a Mother

A strong feeling throughout the survey and interviews was how the experience of not being able to afford formula had affected how they viewed themselves as a mother. Many felt that they were not a ‘good enough’ mother and felt like a ‘failure’.

‘I hope my baby never knows we struggled and that it doesn't leave an impact on her’.

(Survey: mother, age 19–24, White British, baby 6 months, exclusive formula)

For some, this went as far as feelings of regret at having a baby, which caused significant distress and guilt at feeling this way.

‘Sometimes I regret having this baby and I feel awful about having those thoughts because I love her so much but I'm not a good mum to her’.

(Survey: mother, age 19–24, White British, baby 3 months, exclusive formula)

Many described how their early experiences of being a parent had been changed or damaged by their financial situation. A common experience was to feel like they were wishing the time away, or that their maternity leave had been ruined.

‘And I suppose the sad thing is sort of with it at the moment I'm kind of sat here thinking. I can't wait until baby's on solids, because then I don't have to buy as much milk’.

(Interview: Lisa, mother, aged 35–44, White, baby 4 months, exclusive formula)

Stakeholders often described seeing this in the parents they supported.

‘I imagine they would question their ability to be a good enough parent, leading to lack of confidence and feelings of self‐doubt’

(Survey: Infant Feeding Healthcare Support Worker)

### 3.3 Impacts upon infant feeding practices

Parents were then asked what steps they had taken to mitigate the high costs of formula milk, such as using different milks, stretching out feeds or introducing solid foods early. Additionally, stakeholders who worked directly with families (*n* = 123) reported which approaches they had seen themselves or heard about from colleagues. The number who reported ever using/hearing about different approaches is shown in Table 5. Almost half of parents reported sourcing infant formula from social media sites, with 41% introducing solid foods earlier than recommended (47% of those with a baby over 6 months). In terms of specific practices, 39.1% (*n* = 102) reported any unsafe feeding practice (including 28.7% using leftover formula later, 21.5% cutting corners on sterilisation, 17.6% trying to space out feeds, 13.8% diluting infant formula and 8.4% using infant formula past its sell by date. In the interviews and open‐ended boxes, parents expanded on how they coped.

**Table 5:** Ways in which parents mitigate the high costs of formula milk.

Have you ever had to do, or chosen to do, any of the following to be able to afford formula milk for your baby?	Parents						Professionals	
	Full sample		Access healthy/best start		Do not access Healthy/best start			
	N	%	N	%	N	%	N	%
Buy/access formula from somewhere other than a shop, e.g. from Facebook Marketplace or someone you know	120	46.0	49	52.1	71	42.5	68	55.2
Used formula milk aimed at older babies as it was cheaper	118	45.2	58	61.7	60	35.9	27	22.0
Introduced solid foods earlier than recommended to reduce formula costs	108	41.4	40	42.6	68	40.7	66	53.6
Introduced solid foods earlier than recommended to reduce formula costs (parents with a baby aged 6 months+ , *n* = 84)	40	47.6	15	48.4	25	47.2	—	—
Make up several feeds at once and keep them for later	91	34.9	40	42.6	51	30.5	43	35.0
Travel further to find milk at a cheaper price	84	32.2	32	34.0	52	31.1	—	—
Boil less than 1 litre of water when making up feeds to save electricity	84	32.2	42	44.7	42	25.1	24	19.5
Breastfed more or for longer than wanted or advised	81	31.0	33	35.1	48	28.7	30	24.0
Used leftover formula from a feed later	75	28.7	35	37.2	40	24.0	51	41.5
Cut corners on sterilising bottles and other feeding equipment	56	21.5	24	25.5	32	19.2	25	20.3
Try to space feeds out more	46	17.6	27	28.7	19	11.4	33	26.8
Use less formula powder/more water than is recommended when making up a feed to make it go further	36	13.8	19	20.2	17	10.2	71	57.7
Buy a different brand that I may not prefer	33	12.6	19	20.2	14	8.4	—	—
Give cow's milk for some or all feeds instead of formula	28	10.7	8	8.5	20	12.0	35	28.5
Add cereal to formula milk to make it go further	23	8.8	9	9.6	14	8.4	23	18.7
Use formula i.e. past its use by date	22	8.4	10	10.6	12	7.2	16	13.0

Parents who were accessing Healthy/Best Start were significantly more likely to use less formula powder/more water than is recommended when making up a feed to make it go further [*χ* ^2^ = 5.092, *p* = 0.024], boil less than one litre of water when making up feeds to save electricity [*χ* ^2^ = 10.512, *p* = 0.001], try to space feeds out more [*χ* ^2^ = 12.465, *p* = 0.001], used formula aimed at older babies because it was cheaper [*χ* ^2^ = 16.13, *p* = 0.001], Used left over formula from a feed later [*χ* ^2^ = 5.181, *p* = 0.023], and bought a different brand that they did not prefer [*χ* ^2^ = 7.620, *p* = 0.006].

Parents expanded on ways they afforded formula or made it go further. Professionals reflected on the behaviours they had seen or been told about.

#### 3.3.1 Not Following Recommended Feeding Practices

There were several examples in the surveys and interviews of parents deciding to not follow recommended guidance to make milk go further. Some parents described watering down formula milk/using fewer scoops of powder despite knowing that this was potentially harmful and feeling guilty as a result.

‘I've had to use less powder just to be able to, you know, get to a new week, so that I can plan for a new one… The only thing I've done during difficult times is I try to use less powder, and which is really bad. Yeah, I've used less powder before, which is really bad’.

(Interview: Marie, Mother, aged 25–34, Black, baby 12 months, mixed feeding)

Others didn't follow guidance to discard used bottles within the recommended time, to ensure their baby finished the feeds.

‘We have probably fed her from a bottle that shouldn't have. You know, like it's gone over the 2 h limit. So in our minds, we've kind of eked it a little bit and made sure that the bottle that she started, she's finished. Maybe I want to say it's only been an hour on top of it's been a 3 h bottle rather than a 2 h bottle, but I do make sure that they're kept cool’.

(Interview: Alice, mother, aged 25 – 34, White, baby 4 months, exclusive formula)

Stakeholders had also seen these behaviours

‘When they can't access it safely we see the repercussions of that, and that is the unsafe feeding practices of stretching out milk feeds, bulking up milks’.

(Interview: Infant feeding charity)

‘There have been families in their areas that have been struggling with affording infant formula, particularly when the prices had gone up and reports of, you know, families missing feeds or diluting feeds’.

(Interview: Infant feeding organisation)

#### 3.3.2 Sourcing Formula From Online Marketplaces

Parents often responded to adverts on online marketplaces or put out social media requests for formula. They typically collected this from people they did not know, including tubs that were half used.

‘I've managed to pick up a few tubs for free on marketplace, sometimes they're half gone but it's always fine and every bit helps. Friends have given me left over stuff they don't need any more too and I've done a few facebook posts saying we're out and can anyone help. It's hard but I do find I manage by looking about and asking’.

(Survey: mother, age 25–29, White British, baby 2 months, exclusive formula)

This was also reflected in the stakeholder experiences

‘I often see local Facebook posts asking for donations of formula from struggling parents’.

(Survey: Infant feeding specialist)

#### 3.3.3 Using Milks That Are Not Recommended

As shown in Table 5, almost half of parents reported using milks marketed at older babies, with some recognising that these were not recommended but were lower in cost.

‘The milk we use is for babies over 6 months but it's similar and usually on offer so it works out much better and she's always been fine on it’.

(Survey: mother, age 25–29, White British, baby 2 months, exclusive formula)

Stakeholders also reflected on the different milks they had seen used, including an increased attempt to have allergy formula prescribed. Others saw prescription and follow‐on milks donated to food banks or given away on social media marketplaces.

‘Using incorrect formula e.g. if someone on a group is giving away free stage 2 or special milk they may use this’.

(Survey: Breastfeeding counsellor)

There was also an increase in parents asking their GP for prescription formula for babies with cow's milk allergy without obvious symptoms that their baby has an allergy, since this can be accessed for free in the NHS.

‘Asking GP for prescription for cow's milk allergy formula. Therefore it is free. I see many many parents suggesting their baby has CMPA but I cannot see any sign. I have to take their word for it that their baby gets skin rashes and constipation’.

(Survey: General Practitioner)

#### 3.3.4 Introducing Solid Foods Early

As the survey data showed, starting solids early was a common coping strategy. Solid foods were often seen as cheaper/easier to incorporate within a household budget than formula milk was.

‘So we started weaning early because the formula was getting a bit too stressful for us. And then his feeding difficulties as well as the expense. So we started weaning at 3 1/2 4 months’.

(Interview: Leila, mother, aged 25 – 34, Asian, baby 6 months exclusive formula)

Stakeholders had also seen parents resort to early introduction of foods to combat cost

‘There's early weaning, so early introduction of solids. Somebody was saying they're going to give them some baby rice, because the formula is so expensive’.

(Interview: Family health and wellbeing strategy lead)

### 3.4 Impacts and interactions with breastfeeding experience

The survey did not directly ask about breastfeeding experience, but many mothers brought this up in the open‐ended boxes. Most mothers had some experience of breastfeeding including continued mixed feeding, or having initiated but stopped breastfeeding. The influence of breastfeeding experience, financial difficulties formula milk prices were interwoven and complex.

#### 3.4.1 Increased Use of Formula Due to Return to Work

Some mothers made the decision to return to work earlier than planned due to the cost of living. This meant that they often stopped breastfeeding or introduced formula milk earlier than planned.

‘Due to high costs of everything I had to go back to work early (I'm self‐employed) and put my baby in childcare earlier than I wanted. This ironically meant introducing formula as I couldn't express but then we realised how much those costs added up’

(Survey: mother, age 25– 29, White Other, 4 months, mixed feeding)

#### 3.4.2 Worries That Diet Quality Due to Low Income Would Affect Milk

Others were worried about their own diet, which was affected by high food costs and low income would affect the quality of breastmilk, so used more formula.

‘I do have to skip some meals just to balance everything. I don't really see it as a problem now, I've gotten a bit used to it, and I'm just concerned because I need to eat well if I'm also going to breastfeed a child as well. Yes, but most times when I know that my eating for that week wasn't really healthy, I just give her more of the formula milk’.

(Interview: Lucy, aged 25 – 34, Black, baby 7 months, exclusive formula)

#### 3.4.3 Continuing Breastfeeding Due to the Cost of Infant Formula

Several mothers interviewed or surveyed stated that they were breastfeeding more than they wanted to because of the price of formula. This means they were not breastfeeding out of informed choice but as a method of making ends meet.

‘I don't want to be breastfeeding any more but it saves money so feel I have to’.

(Survey: mother, age 25–29, White, baby 4 months, mixed feeding)

These experiences were also reflected in stakeholder experiences

‘Pressure to breastfeed through pain when they might like to stop (although there is the issue of timely support to improve breastfeeding which can be lacking)’.

(Survey: Breastfeeding peer supporter)

#### 3.4.4 The Double Burden of Feeling Let Down With Breastfeeding and Let Down With the Cost of Formula

Many mothers had wanted to breastfeed their baby and had been unable to do so either exclusively or partially, leaving them not feeling supported to breastfeed nor with the cost of infant formula.

‘I always planned to breastfeed but had a lot of pain and found it difficult to get the right advice about what to do and I had to start using formula alongside feeding her myself even though I didn't want to. I'm angry that no one could help me and now no one will help me again because we don't qualify for help as earn that bit too much. I feel we've been let down then and let down again now’.

(Survey: mother, age 30–34, White British, 4 months, mixed feeding)

Stakeholders had also seen this double guilt

‘Feeling guilty and like a failure, twice over if they tried and weren't supported enough to breastfeed’

(Survey: Breastfeeding peer supporter)

#### 3.4.5 Feeling Criticised for ‘Choosing’ to Formula Feed on a Low Income

These feelings were made worse by those who criticised their ‘decision’ to use formula milk when it was an expensive product or suggested they should have tried harder to breastfeed.

‘Someone on social media said that if formula is so expensive that I can't afford it then I should have just breastfed as if it were that simple. I wanted to breastfeed! But I couldn't as she wouldn't latch right. So I feel guilty for that and guilty for not being able to afford the milk she needs. I feel like I've failed her before life has even properly began’.

(Survey: mother, age 35 − 39, White, baby 2 months, exclusive formula)

Stakeholders raised the idea that the high cost of infant formula could be interpreted as punishment for not breastfeeding

‘Like you're being penalised, you can't even afford to feed your baby’.

(Survey: Breastfeeding peer supporter)

#### 3.4.6 Guilt Driven Purchasing

This experience often led to mothers wanting to buy more expensive formula milk brands that they viewed as ‘better’ or ‘best’. When they then also couldn't afford these high prices, this exacerbated feelings of guilt and shame, even though in some cases they were able to afford lower‐priced milks for their baby.

‘I wanted to breastfeed and when I couldn't I wanted to buy the best milk on offer but couldn't afford that and can barely scrape enough together for the cheap ones. This has had a devastating effect on my mental health’.

(Survey: mother, age 35–39, White British, baby 3 months, exclusive formula)

Stakeholders also saw this effect

‘Where I work breastfeeding initiation rates are high so many of these parents may already feel that way for having to give formula at all. Feeding your baby is an absolute basic and to be struggling to do that in whatever way is important to you (including where parents perceive more expensive brands to be “better” despite their unaffordability) is a deeply challenging position to be in’.

(Survey: NHS Infant Feeding Coordinator)

## 4 Discussion

This is the first UK study to highlight in depth the numerous ways in which the high cost of infant formula is impacting upon feeding practices, potentially placing babies at risk of harm, and illustrating the many ways parents try to cope with the cost through reducing their own meals and comfort, and borrowing money. These experiences significantly impact upon parental health, wellbeing and relationships. Whereas families can choose in some cases to buy lower‐priced products or make food swaps depending on food prices, infant formula milk cannot be substituted for another product, illustrating an urgent need for interventions to ensure parents are able to access the formula milk they need for their baby. Together our data show that high formula milk prices are not simply an economic or financial issue but one which ripples out potentially across infant health, parent wellbeing, and family security and relationships.

Our data adds to a body of work identifying issues of financial precarity, that is often unseen or hidden, amongst working families. We included parents who reported struggling to afford infant formula, as against solely parents on a low income. Often, research examining financial difficulties is focussed solely on income rather than income *after* essential costs, however around two thirds of parents in our study were not accessing support via the Healthy/Best Start scheme. Although some were unaware of the scheme, most had an income too high to qualify but were still struggling to afford infant formula due to the increased costs of housing, utilities and childcare alongside impacts of reduced maternity pay. UK data approximates that there are 3.5 million families in the UK with a child under five (ONS 2023). Approximately 10% of these families access Healthy Start (although it is estimated that a third of eligible families do not) (NHS 2026). However, data from the Food Foundation shows that a quarter of households with a child under 5 experience food insecurity (Goudie 2022). A core reason for this disparity is the cost‐of‐living crisis where basic household costs have increased far higher than salaries (Joseph Rowntree Foundation 2024). In the UK, 72% of children living in poverty are from working families (CPAG 2025). Many of these families would be unlikely to qualify for Healthy/Best Start.

This highlights the potential need to consider widening eligibility for access to Healthy/Best Start (or other financial support schemes) and to recognise that issues surrounding formula affordability are not only affecting parents on a gross low income but those with little income remaining after core costs, particularly when receiving maternity pay. Parents who are on a lower income (or low income after core costs) but are not eligible for financial support are increasingly reporting feeling abandoned, stretched and disillusioned (YouGov 2024). Many parents in our research were working, yet facing financial difficulties, exacerbated by low maternity pay.

It also identifies how the amount of Healthy/Best Start allowance does not fully cover the cost of feeding a baby, as some parents were still resorting to methods to make the milk go further due to the broader high cost of living. The allowance has risen from £8.50 to £9.30 per week since we collected our data, but at the time of the study, the allowance did cover the cost of one tub of two of the lowest cost milks on the market per week. However, these products might not be available in a local area, a tub may not last a full week, and there are additional costs on top of the milk itself (e.g. bottles, teats, sterilisation). Others may be using vouchers for wider household food budgets for other family members due to the high costs of food. There is also the issue that misleading marketing drives some parents to feel pressure to purchase more expensive milks out of a belief or anxiety they are better (Brown et al. 2020; Munblit et al. 2020; Rollins et al. 2023). Without stronger regulation of formula milk promotion, increasing allowance may also contribute to increasing industry profits.

There was a clear reported detrimental impact of infant formula affordability upon parent wellbeing, showing this is a wider issue than infant nutrition. Parents typically managed to access formula milk for their baby but to the detriment of their wellbeing, parental identity, relationships and financial security. These experiences have all been shown to exacerbate the risk of postnatal depression (Wang et al. 2021; Sonnenburg and Miller 2021; Taylor et al. 2021). Some mothers continued to breastfeed through pain and difficulty, which has been shown to have a negative impact upon maternal mental health (Brown et al. 2016).

The high cost of infant formula milk also led parents to sometimes adopt behaviours that might put babies at risk such as using milk that was past its sell‐by date, watering down feeds, spacing out feeds or saving used formula for later. These practices reflect those adopted by parents in the recent formula milk shortages in the USA (Sheehan et al. 2025). However, these practices may increase risk of infant illness, poor growth or malnutrition (Scientific Advisory Committee on Nutrition 2018) and go against NHS guidance on safe formula milk preparation due to increased risk of gastrointestinal infection (Crawley et al. 2022; Redmond et al. 2009; Cho et al. 2019). Parents typically were aware that these behaviours were risky and felt guilt and anxiety over doing them.

Sourcing formula from social media sites (including open products) was common with almost half the sample doing this at some point. This was often described in a neutral or positive way, with little awareness of potential risk of bacterial contamination which could lead to gastrointestinal infections in infants (Cho et al. 2019). It may be that for unopened, in date products, there is little risk, but use of products marketed at older babies, specialist formulas or prescription formulas were mentioned by some. Given how widespread this approach is, with variation in safety, there needs to be increased awareness of potential risks and considerations.

Together, these data highlight a strong health inequity and social injustice issue for parents and babies on a low income, particularly mothers (Spencer et al. 2019). Mothers often felt a strong sense of guilt at not being able to breastfeed (typically due to a lack of support) and guilt and anxiety at not being able to then purchase what they were led to believe were the ‘best’ formula milks for their baby. Breastfeeding through pain, feeling forced to use unsafe feeding practices, shop lifting, meeting strangers found online, and constantly thinking about how to source formula, how to make it go further and how to juggle money, work and debt, all carried a significant moral burden and emotional load, especially for women (Callahan 2021). This is particularly unjust when these burdens have been driven by deliberate inaccurate marketing strategies to make parents feel that certain products are better (Hastings et al. 2020).

A further theme was the interaction of poverty and breastfeeding. Many women in our research had planned to breastfeed but had not been supported to do so, meaning they faced a double burden of not breastfeeding and not being able to afford infant formula. This further shows the need to invest in breastfeeding support to enable more women who want to breastfeed to meet their goals, and fewer to have to use formula milk when they do not plan to (Brown 2019). This also affected brand choices. Not being able to breastfeed led to some mothers feeling that they needed to buy more expensive formula milks due to misleading marketing pitching certain brands as closer to breastmilk (Hastings et al. 2020).

Others believed that their breastmilk would be negatively impacted by their own insufficient diet due to food costs. This is a common worry (MacMillan Uribe and Olson 2018), exacerbated by some misleading formula milk marketing (van Tulleken et al. 2020). Although some reviews report a correlation between maternal diet and breastmilk content (Petersohn et al. 2024), many studies have small effect sizes, do not accurately measure dietary intake, do not showing a causal chain between maternal diet, breastmilk content and infant health outcomes (Bravi et al. 2016; Favara et al. 2024), with many implicated by conflicts of interest through breastmilk substitute industry funding (Pereira‐Kotze et al. 2022). However, it is likely that maternal health and wellbeing may suffer is she does not get sufficient nutrients and energy (Lee and Kelleher 2016), again highlighting the importance of ensuring breastfeeding mothers receive financial support.

Together, this shows the need to invest holistically across infant feeding support, rather than considering breastfeeding and formula feeding as two opposing behaviours. We must fix the postcode lottery for community breastfeeding support (Grant et al. 2017) and the broader maternity care crisis in the UK, which disproportionately negatively affects families from low‐income communities (NSPCC & UNICEF 2024).

Finally, use of follow‐on and toddler milks marketed at older babies was also common, typically driven by lower prices or discounts and offers. Follow‐on formulas are unnecessary for 6‐ to 11‐month‐old babies where infant formula is available and should never be used under 6 months (NHS 2023). Toddler formulas are unnecessary for any child and typically contain high levels of sugars (Scientific Advisory Committee on Nutrition 2023). Their promotion also sets up an expectation for a need for formula beyond the point at which it is necessary for infant health (i.e. over 1 year of age), which places unnecessary additional financial burden on parents. This further highlights the need to ensure parents have accurate information on formula milk types and stronger marketing regulation is in place to prevent cross‐promotion and misleading messaging.

Strengths of our research included our mixed methods approach of both survey and interview data from both parents and stakeholders. Our online recruitment approach allowed reach across the UK, was seen as accessible by many participants and did include a range of participants in terms of age, education, ethnicity and income including a third who were accessing Healthy Start allowance. It also identified a group of parents (often with at least one parent working), who are significantly struggling with the cost of living and infant formula prices but do not currently qualify for further support. It is important to draw policy makers' and health care professionals' attention to these families.

Limitations however included a cross‐sectional rather than longitudinal design. Additionally, like many self‐selecting samples, our participant group may be biased to those with the most interest in the topic area and is therefore not representative of all parents on a low income who use formula in the UK. We shared our survey with many community groups, including those supporting refugees and Asylum seekers but did not receive responses from parents from non‐English speaking populations. It is possible that some of the most disadvantaged did not take part potentially due to accessibility but also likely potentially because of fear of perceived reprisals if they discussed not being able to feed their baby or using unsafe feeding practices (Mannay et al. 2018). It is also possible that some parents did not disclose unsafe feeding practices due to social desirability bias, especially in the interviews. A high proportion of mothers in our sample also had experience of ever having breastfed and almost a third were mixed feeding. It is possible that these mothers feel more able to take part in research, as they may feel less criticism because they had tried to breastfeed.

In summary, our study is the first UK based research to highlight the significant issue that high infant formula milk prices combined with the cost‐of‐living crisis are having upon parents and babies. It illustrates the distress parents experience alongside increasing debt and unsafe feeding practices that parents resort to in order to ensure their baby is fed. Given the rapid increase in prices and extensive profits (Competition and Markets Authority 2025), our research illustrates an urgent need for further support and intervention, including enhanced emergency infant feeding pathways, increased investment in breastfeeding support, increased social security payments and wages. Alongside this, government action to strengthen regulations governing the marketing of formula milks and to regulate infant formula prices. This would help to ensure greater infant food security (i.e. access to nutritious foods in sufficient quantities, in the case of infants adding appropriate foods), protecting both infant health and nutrition but wider development and family wellbeing (Zaslow et al. 2009).

## Author Contributions

A.B. was responsible for study conception, study design, data collection, data analysis and draft report writing. C.W. was responsible for study design, data collection, data analysis and draft report writing. V.S., A.G., S.E., S.G., C.G., S.J., H.M., R.P., R.S. and V.T. were responsible for study conception, study design, and draft report writing. All authors read and approved the final manuscript.

## Conflicts of Interest

AB is a volunteer for the Human Milk Foundation and coordinates the Swansea Human Milk Bank hub. SJ is a volunteer for the Human Milk Foundation. VT is a director of First Steps Nutrition Trust. SG is a director of Independent Food Aid Network. SE is a founder of Leicester Mammas. VT is a trustee for First Steps Nutrition Trust and the Medics Lactation community, professional adviser and leader for La Leche League Great Britain, and co‐chairs the Hospital Infant Feeding Network.

## Supporting information


Supporting File 1

Supporting File 2

Supporting File 3

## Data Availability

The data that support the findings of this study are available on request from the corresponding author. The data are not publicly available due to privacy or ethical restrictions. The datasets generated and/or analysed during the current study are not publicly available due to the emotional and personal nature of the full stories given and potentially identifiable information but are available from the corresponding author on reasonable request.
