# Supplementary material for: Infant Formula Affordability Negatively Impacts Parental Wellbeing, Financial Security and Safe Feeding Practices in the UK
Source: Matern Child Nutr. 2026 Jul 31;22(3):e70228. doi: 10.1111/mcn.70228 (PMC13425624; doi:10.1111/mcn.70228)
Supplement: Supplementary file 1 — Supporting File 1 [file MCN-22-e70228-s001.docx]

Start of Block: About you and your baby

The following questions are about your and your baby. We are asking them so we can describe who took part in our research and understand whether experiences of not being able to access formula might be more difficult for some people. Please leave blank any questions you do not wish to answer.

Q1 How old are you?

________________________________________________________________

________________________________________________________________

________________________________________________________________

________________________________________________________________

________________________________________________________________

Q2 What is your ethnicity?

- White - British (English, Welsh, Scottish or Northern Irish) (1)
- White - Irish (2)
- White - Any other White background (3)
- Mixed - White and Black Caribbean (4)
- Mixed - White and Black African (5)
- Mixed - White and Asian (6)
- Mixed - Any other mixed background (7)
- Asian or Asian British - Indian (8)
- Asian or Asian British - Pakistani (9)
- Asian or Asian British - Bangladeshi (10)
- Asian or Asian British - Any other Asian background (11)
- Black or Black British - Caribbean (12)
- Black or Black British - African (13)
- Black or Black British - Any other Black background (14)
- Other Ethnic Groups - Chinese (15)
- Other Ethnic Groups - Any other ethnic group (please describe) (16) __________________________________________________
- Prefer not to say (17)

Q3 What is your sex? (note: A question about gender will follow)

- Male (1)
- Female (2)
- Prefer not to say (3)

Q4 Is your gender the same as your sex registered at birth?

- Yes (1)
- No (if no please enter the term you use to describe your gender) (2) __________________________________________________
- I do not have a gender identity (3)
- Prefer not to say (4)

Q5 What is your highest level of education?

- No formal educational qualifications (1)
- GCSE or equivalent (2)
- A level or equivalent (3)
- Undergraduate degree or equivalent (4)
- Postgraduate degree or equivalent (5)

Q6 Do you have a disability or chronic health issue that affects your day to day living?

- Yes (please add details if you'd like to) (1) __________________________________________________
- No (2)

Q97 Are you neurodivergent (e.g. ADHD, Autism, Dyslexia, Dyspraxia, Tourettes etc)?

- Yes I have a diagnosis (please add details if you'd like to) (1) __________________________________________________
- Yes but I don't have a diagnosis (please add details if you'd like to) (2) __________________________________________________
- Not sure (3)
- No (4)

Q14 Which nation of the UK do you currently live in?

- England (1)
- Wales (2)
- Scotland (3)
- Northern Ireland (4)

Q15 What is the first part of your postcode (this may be 3 or 4 letters and numbers) e.g. SA2 or SA14. We are asking this to understand who we have reached in our survey and also to see if formula poverty is higher in some areas than others.

________________________________________________________________

________________________________________________________________

________________________________________________________________

________________________________________________________________

________________________________________________________________

Q7 Are you currently working?

- Yes full time (1)
- Yes part time (2)
- Yes on a zero hours contract (3)
- Currently on maternity/paternity leave (4)
- No (5)
- Other (please add details) (6) __________________________________________________

Q12 Do you live with a partner? (By partner we mean someone you are married to, in a civil partnership, or live with as if you are married)

- Yes (1)
- No (2)
- Prefer not to say (3)

Q13 If you have a partner are they currently working?

- Yes full time (1)
- Yes part time (2)
- Yes on a zero hours contract (3)
- Currently on maternity/paternity leave (4)
- No (5)
- Other (please add) (6) __________________________________________________

Q11 Please tell us about your relationship to the youngest baby you care for

- I am my baby's mother (1)
- I am my baby's father (2)
- I am a non-birthing co-parent to my baby (3)
- I am a stepparent (4)
- I am a foster parent (5)
- I am a grandparent or other relative who cares for the baby full time (6)
- Other (7) __________________________________________________

Q16 Please tell us the age of your baby or babies 12 months of age and under. If you have more than one baby in this age range you can select more than one answer. If you have twins or triplets etc you can add this detail in the next question.

- Under a month (1)
- 1 month (2)
- 2 months (3)
- 3 months (4)
- 4 months (5)
- 5 months (6)
- 6 months (7)
- 7 months (8)
- 8 months (9)
- 9 months (10)
- 10 months (11)
- 11 months (12)
- 12 months (13)

Q17 How many other children under 18 live in your household? Please include details of step children who may live with you some of the week.

________________________________________________________________

________________________________________________________________

________________________________________________________________

________________________________________________________________

________________________________________________________________

| Page Break |  |
| --- | --- |

Q95 The next few questions ask about your income and outgoings. This is so we can better understand at what level income might affect ability to be able to reliably access / afford formula milk. We've added questions about outgoings because sometimes families can have a higher than average income but costs such as childcare and housing  leave them with little left over. Please leave blank if you do not want to answer. Approximate answers are fine.

Q8 What was the approximate total monthly income of your household last month (after tax and deductions but including any benefits/allowances)?

________________________________________________________________

________________________________________________________________

________________________________________________________________

________________________________________________________________

________________________________________________________________

Q9 What is the approximate amount spent on food and drinks in an average month for your household (this includes food for all members of the household, and items for babies including formula milk)?

________________________________________________________________

________________________________________________________________

________________________________________________________________

________________________________________________________________

________________________________________________________________

Q10 What is the approximate amount spent on bills and essentials in an average month for your household (rent/mortgage, water, council tax, energy, travel costs, childcare)?

________________________________________________________________

________________________________________________________________

________________________________________________________________

________________________________________________________________

________________________________________________________________

| Page Break |  |
| --- | --- |

| Page Break |  |
| --- | --- |

End of Block: About you and your baby

Start of Block: About feeding your baby

For these questions please think about your experience of feeding your youngest baby aged 12 months or under.

Q18 How is your baby currently being fed? Please select all that apply

- Formula milk (1)
- Breast milk (2)
- Solid foods (3)
- Cows milk (4)
- Other drinks (other than water - please describe) (5) __________________________________________________

Q107 How much does your usual brand of formula milk cost each week?

________________________________________________________________

________________________________________________________________

________________________________________________________________

________________________________________________________________

________________________________________________________________

Q20 What sort of formula milk do you feed your baby?

- Powdered milk only (1)
- Mainly powdered, some ready to feed/liquid milk (2)
- About half powdered milk, half ready to feed/liquid milk (3)
- Mainly ready to feed/liquid milk, some powdered (4)
- Ready to feed/liquid milk only (5)

Q21 Does your baby have a prescription formula milk e.g. for cows milk allergy?

- Yes (1)
- No (2)

Q22 Which brand or brands of formula milk do you use?

________________________________________________________________

________________________________________________________________

________________________________________________________________

________________________________________________________________

________________________________________________________________

Q23 What were the reasons for choosing that formula milk? Please tick all that apply

- I used it with other babies (1)
- My family tend to use this one (2)
- A health professional recommended it (3)
- Friends recommended it (4)
- It was the one given in hospital (5)
- The ingredients (6)
- The cost (7)
- It's easy to get hold of (8)
- No particular reason (9)
- Another reason (please describe) (10) __________________________________________________

| Page Break |  |
| --- | --- |

Q24 Do you ever struggle to afford the formula you need to buy for your baby?  By 'struggle' we have you ever found yourself struggling to afford to buy formula milk but managed to do so by making significant cuts elsewhere such as going without food yourself, not being able to heat your home, not paying a bill, taking on extra work or getting into debt? Or you've had to ask for help (from family, friends, a health professional, food bank or charity) so your baby doesn't go without?.

Although difficult, for this survey we don't mean having to making smaller changes to your budget such as cutting back on things you'd like to buy in order to afford it (even though we recognise formula is very expensive).  If you have never struggled to afford formula, or managed to afford it by making small changes you'll be taken to the end of the survey and we very much appreciate the time taken to add your responses so far as they help us understand the bigger picture. Thank you.

- Yes (1)
- No - I can afford formula milk without having to cut back (2)
- No - I can afford formula milk by making smaller changes to my budget (6)

Start of Block: About the cost of living crisis and your household

| Page Break |  |
| --- | --- |

Q32 Since your baby has been born, have you ever been referred to a food bank? Please select all that apply

- Yes and I went to the food bank (1)
- Yes, I have been referred but I did not go (3)
- No, I needed to but haven't asked as I didn't think I would quality (5)
- No, I needed to but didn't think I would be able to get there (10)
- No, I asked but was unable to get a referral (4)
- No, I needed to but didn't know how to access one (8)
- No, I didn't realise it was an option (7)
- No, but I have been in the past (6)
- No, I didn't need to (11)
- No, other (please describe) (9) __________________________________________________

Q33 If yes, how many times have you been able to access a food bank in the past year?

________________________________________________________________

________________________________________________________________

________________________________________________________________

________________________________________________________________

________________________________________________________________

Q34 Who referred you to a food bank (e.g. health visitor rather than a name)?

________________________________________________________________

________________________________________________________________

________________________________________________________________

________________________________________________________________

________________________________________________________________

| Page Break |  |
| --- | --- |

The next questions ask about your experiences of affording formula milk for your baby. We are asking them so that we can show the impact that the cost of living crisis and cost of formula milk is having on parents.

Q36 Have you ever found yourself struggling to afford to buy formula milk to the point that you've had to ask for help (from family, friends, a health professional, food bank or charity etc) so your baby doesn't go without? If so, how often does this happen?

- All the time (1)
- Weekly (2)
- A couple of times a month (3)
- Monthly (4)
- Every few months (5)
- A couple of times a year (6)
- Once a year or less (7)
- Never (8)

Q37 Have you ever found yourself struggling to afford to buy formula milk but managed to do so by making cuts elsewhere such as going without food yourself, not being able to heat your home, not paying a bill, yourself or your partner taking on extra work or borrowing money? If so, how often does this happen?

- All the time (1)
- Weekly (2)
- A couple of times a month (3)
- Monthly (4)
- Every few months (5)
- A couple of times a year (6)
- Once a year or less (7)
- Never (8)

Q105 If you've made cuts / changes to be able to afford formula milk for your baby what have you or your partner (if applicable) done? Please tick all that apply

- Not heated our home as much as we needed (1)
- Gone without meals myself (2)
- Not paid bills on time (3)
- Borrowed money that is difficult to pay back (4)
- Sold important items (5)
- Gone back to work from maternity / paternity leave early (6)
- Taken on more work (over and above typical full time hours) (8)
- Gone without basic new clothing for myself (7)
- Sold / stopped using a car (9)
- Gone without things like opticians appointments, prescriptions, check ups (10)

Q106 Anything else not described above?

________________________________________________________________

________________________________________________________________

________________________________________________________________

________________________________________________________________

________________________________________________________________

Q35 If you are also breastfeeding, have you felt under pressure to keep giving your baby breastmilk when you might want to or need to stop because of the cost of formula?

- Strongly agree (1)
- Agree (2)
- Neither agree nor disagree (3)
- Disagree (4)
- Strongly disagree (5)
- I'm not breastfeeding (6)

Q38 How does struggling to afford to be able to buy formula milk make you feel?

________________________________________________________________

________________________________________________________________

________________________________________________________________

________________________________________________________________

________________________________________________________________

Q40 I feel that not being able to afford formula milk has negatively affected my

|  | Strongly agree (42) | Agree (43) | Neither agree nor disagree (45) | Disagree (47) | Strongly disagree (48) | Not applicable to me (49) |
| --- | --- | --- | --- | --- | --- | --- |
| Mental health (1) |  |  |  |  |  |  |
| Physical health (2) |  |  |  |  |  |  |
| Relationship with my partner (3) |  |  |  |  |  |  |
| Relationship with my baby or children (4) |  |  |  |  |  |  |
| Ability to relax and enjoy life (5) |  |  |  |  |  |  |
| Ability to concentrate on work or other responsibilities (6) |  |  |  |  |  |  |
| Time on maternity / paternity leave (7) |  |  |  |  |  |  |
| How I feel about myself as a new parent (8) |  |  |  |  |  |  |

Q41 Please tell us about any other ways you feel it might have affected your life

________________________________________________________________

________________________________________________________________

________________________________________________________________

________________________________________________________________

________________________________________________________________

| Page Break |  |
| --- | --- |

The next few questions ask about the costs of feeding your baby

Q43 How much do you think it would need to cost for you to be able to more easily afford it each week? If you feel you cannot afford it still at a lower cost please put '£0'

________________________________________________________________

________________________________________________________________

________________________________________________________________

________________________________________________________________

________________________________________________________________

Q44 When I think about the overall cost of feeding my baby I worry about the cost of (please select all that apply)

- The formula milk (1)
- Getting to the shop to buy it (2)
- Bottles and teats (3)
- Cleaning and sterilising (4)
- Electricity costs to prepare it (5)
- Other (please describe) (6) __________________________________________________

Q45 Have you ever had to do, or chosen to do, any of the following to be able to afford formula milk for your baby? We are asking these questions to show the impact formula costs may be having on parents. Please select all that apply

- Buy a different brand that I may not prefer (14)
- Travel further to find milk at a cheaper price (15)
- Use formula that is past its use by date (1)
- Buy formula from somewhere other than a shop, for example from Facebook Marketplace or someone you know (2)
- Use less formula powder/more water than is recommended when making up a feed to make it go further (3)
- Give your baby cow's milk for some or all feeds instead of formula (4)
- Add cereal to formula milk to make it go further (5)
- Boil less than 1 litre of water when making up feeds to save electricity (6)
- Make up several feeds at once and keep them for later (7)
- Cut corners on sterilising bottles and other feeding equipment (8)
- Try to space feeds out more (9)
- Introduced solid foods earlier than recommended to reduce formula costs (10)
- Breastfed more or for longer than you wanted to or were advised to, to reduce formula costs (11)
- Used formula milk aimed at older babies as it was cheaper (12)
- Used left over formula from a feed later (13)

Q48 Is there anything else that you've done to save on the cost of formula feeding that you'd like to add?

________________________________________________________________

________________________________________________________________

________________________________________________________________

________________________________________________________________

________________________________________________________________

| Page Break |  |
| --- | --- |

Q47 Are you aware of the Healthy Start scheme (in England, Wales and Northern Ireland) or Best Start scheme (in Scotland)?

- Yes (1)
- No (2)

Q96 Have you used the scheme?

- Yes, I use it (1)
- Yes, I used to use it, but I don't anymore (2)
- I know about it but I haven't looked into it (3)
- I know about it but I don't think I'd be eligible (4)
- I know about it but I don't know how to access it (5)
- I'm not eligible (6)

If you are not aware of the Healthy Start voucher scheme, if you live in England, Wales or Northern Ireland and you’re more than 10 weeks pregnant or have a child under 4, you may be entitled to get help to buy healthy food and milk. If you’re eligible, you would be sent a Healthy Start card with money on it that you can use in some UK shops. The government would add your benefit onto this card every 4 weeks. You can use your Healthy Start card to buy: plain liquid cow’s milk fresh, frozen, and tinned fruit and vegetables fresh, dried, and tinned pulses infant formula milk based on cow’s milk You can also use your card to collect Healthy Start vitamins – these support you during pregnancy and breastfeeding vitamin drops for babies and young children – these are suitable from birth to 4 years old. Best Start Foods is a payment for people living in Scotland that can help you buy healthy foods like milk or fruit during pregnancy and when your child is under 3. Best Start Foods is paid every 4 weeks. The payments are: £21.20 during pregnancy (you’ll get the same amount whether you’re having one or more babies) £42.40 for each child from birth until they’re one year old £21.20 for each child aged between one and 3 years old If your baby’s born early, you’ll get the higher amount from birth until 1 year after the due date. Please ask your midwife or health visitor whether you might be eligible for one of these schemes.

Q49 How do you pay for/ access your baby's formula if not from household wages or benefits? Please select all that apply now or in the last year.

- Healthy Start / Best Start foods scheme vouchers (5)
- Vouchers from another scheme or charity (6)
- Money given or lent to me by family or friends (8)
- Formula given to me by a foodbank, baby bank or community fridge (9)
- Gift / borrowed from family or friends (10)
- I get it on prescription (11)
- A giveaway from someone I don't know such as on social media or marketplaces (13)
- I've borrowed money e.g. on credit cards (18)
- I've tried to get free samples from formula companies (19)
- I've taken it without paying from a shop (14)
- Other (please describe) (15) __________________________________________________

Q113 How do you pay for/ access your baby's formula if not from household wages or benefits? Please select all that apply now or in the last year.

- Healthy Start / Best Start foods scheme vouchers (5)
- Vouchers from another scheme or charity (6)
- Money given or lent to me by family or friends (8)
- Formula given to me by a foodbank, baby bank or community fridge (9)
- Gift / borrowed from family or friends (10)
- I get it on prescription (11)
- A giveaway from someone I don't know such as on social media or marketplaces (13)
- I've borrowed money e.g. on credit cards (18)
- I've tried to get free samples from formula companies (19)
- I've taken it without paying from a shop (14)
- Other (please describe) (15) __________________________________________________

Q50 Would you like to add any details on how you've paid for or been given formula milk?

________________________________________________________________

________________________________________________________________

________________________________________________________________

________________________________________________________________

________________________________________________________________

Q51 Since your baby has been born have you ever been referred to a food bank and when there asked for formula milk? Please tick all that apply

- I haven't used a food bank (1)
- I used a foodbank but didn't ask for formula because I didn't need it (2)
- I used a foodbank but didn't ask for formula because I knew they didn't have it (3)
- I used a foodbank and was told they didn't have any (4)
- I used a foodbank and was told they weren't allowed to have any (5)

| Page Break |  |
| --- | --- |

The next questions look at options for accessing formula milk when you can't afford it. We fully support what's known as a 'cash first' approach where benefits, wages and allowances are increased and there is more awareness of how to claim support, so you are less likely to struggle to buy formula milk in the first place. However even if this were to happen we think there needs to be a short term or emergency option for families who find themselves in financial crisis. Applying for benefits can take time when babies need to be fed now. The next questions therefore ask how you think parents should be able to access formula if they need it.

Q52 Which of the following options would make it easier for you to get the infant formula you need for your baby when you can't afford to buy it? Please choose as many as you like

- Foodbanks giving out formula (1)
- Food banks giving out vouchers to buy formula (2)
- Community fridges stocking formula (5)
- Healthcare professionals like midwives or health visitors giving out formula (6)
- Healthcare professionals giving out vouchers to buy formula (7)
- Charities or parenting groups giving out formula (11)
- Charities or parenting groups giving out vouchers to buy formula (12)
- The value of Healthy/Best Start vouchers increasing and having wider access to cover the costs of formula feeding a baby (8)
- Increases to benefits /wage increases so I could afford to feed my baby in the first place (9)
- Formula being more affordable in the first place e.g. set lower pricing (10)
- Shops being allowed to put offers on formula (13)
- Shops being allowed to offer loyalty points/ use points or vouchers to buy formula (14)

Q100 If there had to be just one option, which would be your preferred option?

- Foodbanks giving out formula (1)
- Food banks giving out vouchers to buy formula (2)
- Community fridges stocking formula (5)
- Healthcare professionals like midwives or health visitors giving out formula (6)
- Healthcare professionals giving out vouchers to buy formula (7)
- Charities or parenting groups giving out formula (11)
- Charities or parenting groups giving out vouchers to buy formula (12)
- The value of Healthy/Best Start vouchers increasing and having wider access to cover the costs of formula feeding a baby (8)
- Increases to benefits /wage increases so I could afford to feed my baby in the first place (9)
- Formula being more affordable in the first place i.e. set lower pricing (10)
- Shops being able to put offers on formula (13)

Q111 If shops were allowed to offer discounts or loyalty points on formula milk, or you could use loyalty points or vouchers to buy formula, do you feel there would be any benefits or limitations to this?

|  | Strongly agree (1) | Agree (2) | Neither agree nor disagree (3) | Disagree (4) | Strongly disagree (5) |
| --- | --- | --- | --- | --- | --- |
| It would take a long time to accumulate enough points to make a difference (1) |  |  |  |  |  |
| It wouldn't make that much difference in the grand scheme of things as I can spend points on other items (2) |  |  |  |  |  |
| Offers might not be available for my preferred formula (3) |  |  |  |  |  |
| Offers might not be available when I needed them (4) |  |  |  |  |  |
| It would feel more equal i.e. I can buy / receive points for other items so why not formula (5) |  |  |  |  |  |
| It would help me occasionally in an emergency e.g. as a back up (6) |  |  |  |  |  |
| It would feel less stigmatising (7) |  |  |  |  |  |
| A lower set price in the first place would feel more reliable (8) |  |  |  |  |  |
| Offers might be used to encourage purchase of more expensive products when these are not necessary (10) |  |  |  |  |  |
| Companies don't use offers to reduce prices for the consumer - they use it to increase sales so parents wouldn't benefit in the long run (11) |  |  |  |  |  |

Q112 Would you like to add any other thoughts around offers or loyalty points?

________________________________________________________________

________________________________________________________________

________________________________________________________________

________________________________________________________________

________________________________________________________________

Q53 If you were to receive formula at no cost / a voucher to exchange for formula at no cost in an emergency which would be your preferred option?

- Being given formula (1)
- Being given a voucher for formula (2)
- I don't mind (3)

Q54 And who would you prefer to receive formula/ a voucher from?

- A food bank (1)
- A health professional (2)
- A charity or parenting group (3)
- I don't mind (4)

Q108 If you were given a voucher would you prefer to:

- Go to a person to collect it (1)
- Sign up online without seeing/speaking to a person (2)
- I don't mind (3)

Q98 When accessing formula would you like to be able to access any other support at the same time if you needed it? Please tick all that apply

- No, I'd just want to be given the formula/voucher (1)
- Support with feeding my baby (2)
- Support with making sure I'm accessing all benefits / financial support that I can (3)
- Wider parenting support (4)
- Wellbeing support e.g. how difficult it can feel not to be able to access formula (5)

Q99 When accessing formula, how important would it be to you that the person giving you the voucher / support had training in supporting parents and feeding so that you could ask other questions if you needed to?

- Very important (1)
- Fairly important (2)
- A little important (3)
- Not important at all (4)

Q55 Would you like to explain your reasons for your choices?

________________________________________________________________

________________________________________________________________

________________________________________________________________

________________________________________________________________

________________________________________________________________

Q56 One solution is to have donations of infant formula or vouchers available at food banks. If this was an option, would you see any benefits in this?

- Being able to pick it up when I was already there if I had a referral (12)
- Feeling supported in how I feed my baby because it was available (2)
- Formula being treated like any other food (13)
- Other (please add details) (11) __________________________________________________

Q57 Do you think there are any possible difficulties to accessing formula milk / a voucher via a food bank if this was the main way to receive it?

- Travelling to the food bank to get it (12)
- Getting to the food bank when they are open (1)
- Needing it urgently when the food bank is not open (2)
- Not qualifying for a food bank referral but not being able to afford formula milk (6)
- I don't want to ask for a food bank referral - I just need the formula milk (10)
- That I would be judged for asking for it (13)
- Other (please add details) (11) __________________________________________________

Q101 If food banks accepted donations of infant formula to distribute, apart from the concerns above, would you worry about any of the following?

- The brand my baby usually drinks would not be offered (8)
- It being the wrong 'type' e.g. not for babies under 12 months (7)
- Not knowing where it had come from e.g. who donated it (5)
- It might not always be available when I needed it because food banks rely on donations (9)
- Other (please add details) (11) __________________________________________________

Q58 Another option is to have infant formula / a voucher available via your health visitor, a parenting charity or similar. If this was an option, would you see any benefits in this?

- Being able to pick it up from somewhere I already go to (12)
- Feeling supported in how I feed my baby because it was available (2)
- Trust in the product because it came from a health service / a charity (13)
- Knowing I could ask questions about feeding my baby if I had them (14)
- Already having a relationship with them e.g. with a health visitor (15)
- Other (please add details) (11) __________________________________________________

Q102 If infant formula rather than a voucher was available via your health visitor or similar would you worry about any of the following?

- Not being able to get an appointment when I need one (6)
- I don't want to ask my health professional for help (10)
- I haven't told my health visitor that I'm using formula (14)
- I don't want my health professional to know I'm struggling (13)
- I'm worried that my health visitor would judge me (15)
- Other (please add details) (11) __________________________________________________

Q103 If health visitors / charities offered tubs of infant formula rather than a voucher, apart from the concerns above, would you worry about any of the following?

- The brand my baby usually drinks would not be offered (8)
- It being the wrong 'type' e.g. not for babies under 12 months (7)
- Not knowing where it had come from (5)
- It might not always be available when I needed it (9)
- Other (please add details) (11) __________________________________________________

Q60 If infant formula was made more affordable via an increase or wider access to Healthy/Best Start allowance would you consider any of the following be a benefit?

- I could just shop as usual (12)
- I can make my own decision on brand (1)
- I can buy it when I need it (2)
- I don't need to make an appointment (13)
- It puts me in control (14)
- I don't have to ask a person for help (15)
- It feels less judgemental - I'm trusted to make my own decisions (16)
- It feels fair that the full cost is covered (17)
- Other (please add details) (11) __________________________________________________

Q61 If infant formula was made more affordable via an increase or wider access to Healthy/Best Start allowance would you worry about any of the following?

- Needing it urgently but it taking time to apply (1)
- Needing it but being told I didn't qualify (2)
- Not knowing how to apply (4)
- Struggling with filling in forms or providing evidence (5)
- Shops not accepting the voucher/allowance (6)
- The brand my baby usually drinks would not be covered (7)
- I don't want to ask for help this way (8)
- Other (please add details) (9) __________________________________________________

Q62 If you have any other ideas about what would make it easier for you to access the formula you need to feed your baby, please tell us below

________________________________________________________________

________________________________________________________________

________________________________________________________________

________________________________________________________________

________________________________________________________________

End of Block: About the cost of living crisis and your household

Start of Block: Further help with our research

This is the end of the survey, thank you for your time. If you have any questions or concerns about feeding or caring for your baby, please contact your health visitor or GP. If you are worried about your own wellbeing you may also like to contact your health visitor or GP or may find additional support from Mind The Samaritans  The Maternal Mental Health Alliance  NHS mental health services near you You can find more information about Healthy Start and Best Start here  If you have any questions or concerns about accessing food for yourself or your family or other financial or housing concerns you can access support through Fare Share

End of Block: Further help with our research
