# Supplementary material for: Infant Formula Affordability Negatively Impacts Parental Wellbeing, Financial Security and Safe Feeding Practices in the UK
Source: Matern Child Nutr. 2026 Jul 31;22(3):e70228. doi: 10.1111/mcn.70228 (PMC13425624; doi:10.1111/mcn.70228)
Supplement: Supplementary file 2 — Supporting File 2 [file MCN-22-e70228-s003.docx]

Q1 Please tell us about your involvement with food insecure parents. You can select all that apply.

- I am a health or social care professional (1)
- I am a paid peer worker (2)
- I am a volunteer (3)
- I have a non parenting facing role such as a manager, policy specialist or strategic lead (4)

Q2 My work / volunteering is based around (please select all that apply)

- Infant feeding support (1)
- Parenting support (2)
- Food bank support (3)
- Baby bank support (4)

Q3 Please enter your job role/title (if you would prefer to leave this blank if it identifiable please do so)

________________________________________________________________

Q4 Does your role directly involve supporting parents face to face?

- Yes (1)
- No (2)

Q9 How would you describe the area in which you work?

- Rural – in the countryside and not close to local amenities (1)
- Small town – some local amenities accessible (2)
- Suburban – close to a city or large town with enough amenities accessible (3)
- Urban – in a large town or city with lots of amenities accessible (4)
- My role crosses areas / is national (5)

Q5 Where do you support parents? (select all that apply)

- NHS premises (1)
- In community premises (e.g. children’s or community centres, schools) (2)
- At a food bank (3)
- At a baby bank (4)
- In parents’ homes (5)
- I don't see parents face to face (7)
- Other (6) __________________________________________________

Q10 To give us an idea of the demographic group you support, please tell us how you view the area you work in

- Affluent (1)
- Deprived (2)
- A mixture of affluence and deprivation (3)

Q6 Does your service directly assist food insecure parents/caregivers to access infant formula?

- Yes (1)
- No, it's not within our remit (2)
- No, it's our policy not to do this (3)
- No, we are not parent facing (4)

Q7 If yes, what kind of help does it provide? (Please select all that apply)

- My service gives out infant formula directly on demand (1)
- My service gives cash to be spent specifically on infant formula (2)
- My service gives out vouchers that can only be spent on infant formula (3)
- My service directly purchases infant formula for specific families in need (4)
- My service gives cash or vouchers that can be used to afford food more broadly (which could be spent on formula or other essential foods) (5)

Q8 Which country do you work in?

- England (1)
- Wales (2)
- Scotland (3)
- Northern Ireland (4)
- My role crosses countries e.g. a national role (5)

Q11 Have you received training in any of the following issues around infant feeding? Please select all that apply

- The benefits of breastfeeding for babies and mothers (1)
- Why breastfeeding might be important to mothers (2)
- The practical aspects of breastfeeding (e.g. milk supply, latching, frequency of feeds) (3)
- The challenges families can experience with breastfeeding (4)
- How breastmilk supply can be impacted by using formula (5)
- The practical aspects of making up formula safely (6)
- How to support parents to choose a formula milk suitable for their baby (7)
- How formula company advertising can undermine breastfeeding (8)

Q12 How confident and knowledgeable do you feel in your knowledge of breastfeeding and supporting parents ? Please rate yourself on the scale below, if 0 represents no knowledge or confidence on this topic, and 10 represents feeling very well informed and confident on this topic

|  | 0 | 1 | 2 | 3 | 4 | 5 | 6 | 7 | 8 | 9 | 10 |
| --- | --- | --- | --- | --- | --- | --- | --- | --- | --- | --- | --- |

| How confident and knowledgeable do you feel in your knowledge of breastfeeding and supporting parents? () | 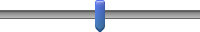 |
| --- | --- |

End of Block: About you

Start of Block: Demand for help in accessing infant formula

Q43 For these next questions we'd like you to think about the topic of accessing infant formula in financial crisis. By this phrase we mean families who are struggling to be able to purchase or access formula at all for their baby, whether this is a very regular occurrence or less often. These families are in the position where they reach out for help because they have no formula available and don't know how they will feed their baby, rather than the many families right now who are finding it an expensive purchase within their budget.

Q49 Do you regularly see parents face to face in your role? This question will make sure the next questions are relevant to you.

- Yes (1)
- No (2)

Q16 If you don't see parents face to face in your service, but hear about the issue of formula insecurity from those working with parents or via other sources such as research or the media, what proportion of families in the UK who use formula milk would you estimate are struggling to afford it to the extent that they often risk running out?

- All of them (1)
- 90% (7)
- Around 75% (2)
- Around half (3)
- Around 25% (4)
- 10% (8)
- None at all (5)
- I don't know (6)

Q13 How frequently are you personally asked by parents for help or support with accessing infant formula in financial crisis?

- Never (1)
- Every 4-6 months (2)
- Every 2-3 months (3)
- Every week (4)
- Every day (5)
- I don't know/we don't discuss this (6)
- Other (please describe) (7) __________________________________________________

| Page Break |  |
| --- | --- |

Q14 Are you aware of other people in your organisation being asked for support by parents in accessing infant formula?

- No one has mentioned it (1)
- People mention it occasionally (2)
- I hear about this regularly (3)
- I hear about this very regularly - it's a significant problem (9)

Q15 Approximately what proportion of parents who use formula milk that you see in your service do you think are struggling to afford to it to the extent that they often risk running out?

- All of them (1)
- 90% (8)
- Around 75% (2)
- Around half (3)
- Around 25% (4)
- Very few or none at all (5)
- 10% (9)
- I don't know (6)

Q65 Do you think this need has increased in recent years?

- Increased a lot (1)
- Increased a little (2)
- About the same (3)
- Decreased a little (4)
- Decreased a lot (5)

Q17 Have you experienced or been made aware of parents who are struggling to buy formula milk doing any of the following? Please select all the apply

- Using formula that is past its use by date (1)
- Buying formula from somewhere other than a shop, for example from Facebook Marketplace or someone they know (4)
- Using less formula powder/more water when making up a feed to make it go further (5)
- Giving cow's milk for some or all feeds instead of formula (6)
- Adding cereal to formula milk to make it go further (7)
- Boiling less than 1 litre of water when making up feeds to save electricity (i.e. not following NHS recommendations on formula preparation) (8)
- Making up several feeds at once and keeping them for later (because of preparation costs) (9)
- Not sterilising bottles and other feeding equipment as per guidance (10)
- Trying to space feeds out more to use less formula (11)
- Introducing solid foods earlier than recommended to reduce formula costs (12)
- Breastfeeding more or for longer than wanted to reduce formula costs (13)
- Breastfeeding when not advised (or not supplementing with formula) to avoid formula costs (17)
- Using formula milk for older babies as it was cheaper/ on offer (14)
- Using left over formula from a feed later (15)
- Taking formula from a shop without paying (16)

Q50 What proportion of parents approximately who are struggling to buy formula milk do you think do at least one of the things above to feed their baby at least some of the time?

- All of them (1)
- 90% (7)
- 75% (3)
- 50% (4)
- 25% (5)
- 10% (8)
- None of them (6)

Q44 Are there any other coping strategies or behaviours that you're aware of that you'd like to add?

________________________________________________________________

________________________________________________________________

________________________________________________________________

________________________________________________________________

________________________________________________________________

Q18 Do you feel that the cost of living crisis and formula costs can negatively impact on some parents:

|  | Strongly agree (1) | Agree (2) | Neither agree nor disagree (3) | Disagree (4) | Strongly disagree (5) |
| --- | --- | --- | --- | --- | --- |
| Mental health (1) |  |  |  |  |  |
| Physical health (4) |  |  |  |  |  |
| Relationship with their partner (5) |  |  |  |  |  |
| Relationship with their baby (6) |  |  |  |  |  |
| Ability to enjoy life as a parent (18) |  |  |  |  |  |
| Ability to feel confident as a parent (19) |  |  |  |  |  |

Q45 How do you feel being unable to afford formula makes parents feel?

________________________________________________________________

________________________________________________________________

________________________________________________________________

________________________________________________________________

________________________________________________________________

Q53 The next questions ask about how parents should be able to access formula if they are experiencing financial crisis.  We fully support a 'cash first' approach where financial crisis is avoided in the first place for parents through sufficient financial support through benefits, work, and sufficient allowances such as Healthy/ Best Start so that they can afford to feed their baby. However even if available this approach can take time to register for and other options may serve parents better, at least in the short term or in an emergency whilst they are given support to maximise income.  The next questions explore some of those different options.

Q63 When parents need to access infant formula in a financial crisis, do you think the person/ service providing formula (or a voucher for formula) should also be trained in offering parents

|  | Strongly agree (1) | Agree (2) | Neither agree nor disagree (3) | Disagree (4) | Strongly disagree (5) |
| --- | --- | --- | --- | --- | --- |
| Feeding support (1) |  |  |  |  |  |
| Parenting support (2) |  |  |  |  |  |
| Mental health support (3) |  |  |  |  |  |
| Support with maximising income e.g. benefits and other sources (4) |  |  |  |  |  |
| None of the above - they should just be given the formula they need (5) |  |  |  |  |  |

Q64 Do you think if parents are financially struggling to the point of not being able to access formula milk for their baby then ideally they should be signposted to their health professional so that services are aware and they can be offered wider support?

- Strongly agree (1)
- Agree (2)
- Neither agree nor disagree (3)
- Disagree (4)
- Strongly disagree (5)

Q47 The next questions explore your thoughts on whether you think that food banks should accept donations of formula milk for parents in financial crisis to access.

Q20 Many food banks in the UK do not currently have infant formula milk available. What is your understanding of why food banks often set a policy of not receiving or distributing infant formula? Please select as many options as you would like

- It's against UK law/legislation (1)
- It's not clear whether it's against the law or not so food banks err on the side of caution (2)
- It's against Department of Health and Social Care rules (3)
- It's against Local Authority rules (4)
- It goes against the UNICEF Baby Friendly Initiative guidance (5)
- I didn't realise there were policies against receiving formula donations (6)
- They think they have to follow Baby Friendly guidelines to keep accreditation of local services (11)
- They want to follow guidance because it's written by people who have expertise on the issue (24)
- Donations aren't reliable / consistent (25)
- They worry that formula donations would undermine breastfeeding (26)
- It's not the best place for parents in need to access formula (27)
- It's just accepted as something that isn't done (28)
- They don't want to take responsibility for babies being fed - the government or health board should do this (29)
- It's too much responsibility for staff (30)
- It would require more training / knowledge (31)
- I don't know (20)
- I've never really thought about why (21)

Q60 Are there any other reasons you'd like to add?

________________________________________________________________

________________________________________________________________

________________________________________________________________

________________________________________________________________

________________________________________________________________

End of Block: Demand for help in accessing infant formula

Start of Block: About food banks and formula distribution

Q21 Some people argue that infant formula should be provided by food banks, but others have voiced concerns about this suggestion. Broadly speaking, taking into account the balance of any concerns you might have, how much are you in favour of infant formula being provided by food banks?

- I am very much in favour of it (1)
- I am somewhat in favour of it (2)
- I am neither in favour or against it (3)
- I am somewhat against it (4)
- I am definitely against it (5)

Q22 Can you see any benefits to food banks providing infant formula to food insecure parents?  (please select all that apply - a question on risks will follow)

- Parents are already coming to food banks for food for their family, so it makes sense to provide formula at the same time (1)
- It would reach those parents most in need (2)
- Infant formula should be free, and this is the best way to implement that principle (3)
- I would have concerns about cash or vouchers give for formula being spent on other things (4)
- It gives donors/the public the chance to help vulnerable families (5)
- It feels like a non judgemental option (6)
- Formula is a food for babies and food banks provide food, so it makes no sense not to have it (7)

Q23 Are there any other potential benefits you can think of for formula being available at food banks?

________________________________________________________________

Q24 Can you see any risks to food banks providing infant formula to food insecure parents? (please select all that apply)

- It’s not reasonable to expect food bank staff/volunteers to know enough about different types of formula to ensure the right product reaches each family (1)
- Donations could be too patchy or inconsistent (2)
- Donations could be inappropriate (i.e. prescription only, toddler milks, close to use by dates) (3)
- It undermines parental choice in terms of what brand of infant formula to feed their baby (4)
- Not everyone can get a referral to a food bank (5)
- Not everyone can easily travel to a food bank (6)
- Obtaining formula in this way misses out the vital step of ensuring parents know how to prepare it safely (7)
- I would worry it would undermine breastfeeding because parents would be encouraged to take it (8)
- If families are struggling to feed their baby I would want them to have support with feeding too (9)
- It could allow formula companies to donate milk with the aim of increasing sales (10)
- We should be increasing financial benefits not handing out products (11)

Q25 Are there any other risks you can see?

________________________________________________________________

Q26 If you work/volunteer in a food bank, how happy (e.g. feeling knowledgeable,confident and able to do this)  would you be giving out formula to food insecure parents if asked to by your organisation?

- Completely happy (1)
- Fairly happy (with a few concerns) (2)
- Somewhere in the middle (3)
- Fairly unhappy (with many concerns) (4)
- Very unhappy (5)
- N/A - I dont work/volunteer in a food bank (6)

Q62 If you would have concerns about giving out formula, what would these be? Please choose all that apply

- It goes against the UNICEF Baby Friendly guidance (1)
- It might undermine support for breastfeeding (2)
- We wouldn't have the right stock for individual families (3)
- We might not be able to offer a consistent supply (4)
- Even though my organisation says it's OK I would still be breaking rules (5)
- I just don't have enough training around infant feeding to support/advise parents about baby milk (6)
- I don't think we should be the place parents go to - they should see a health professional (7)
- Families would come to rely on us / expect it to be here (8)
- I think parents in that situation need lots of support and I don't have time to chat (9)
- It lets the government off supporting parents directly e.g. increasing benefits or allowances so that they can afford formula in the first place (10)

Q51 Are there any other concerns you'd like to add?

________________________________________________________________

________________________________________________________________

________________________________________________________________

________________________________________________________________

________________________________________________________________

End of Block: About food banks and formula distribution

Start of Block: Voucher schemes for purchasing infant formula

Q52 As described above we fully support a 'cash first' approach where financial crisis is avoided in the first place for parents through sufficient financial support through benefits, work, and sufficient allowances such as Healthy/ Best Start. However even if available this approach can take time to register for and other options may serve parents better, at least in the short term. Another one of those options is having vouchers available that parents can access that can be exchanged for formula in shops. These might be available from charities, health professionals, local authorities or other supporter organisations. The next questions explore your opinions on these as an option.

Q30 Please tell us how in favour of a voucher scheme you would be

- I am very much in favour of it (1)
- I am somewhat in favour of it (2)
- I am neither in favour or against it (3)
- I am somewhat against it (4)
- I am definitely against it (5)

Q31 Can you see any benefits to a voucher scheme for providing infant formula to food insecure parents? Please select all that apply

- You can be sure that the voucher is definitely used for formula (2)
- It might feel less stigmatising than attending a food bank or baby bank (3)
- It’s easy to keep track of what has been given out (4)
- It doesn’t affect food insecure parents’ state benefits (5)
- It might be a quicker way for parents to access formula (6)
- It would be a more reliable way to access formula e.g. not relying on donations being present (7)
- Some parents might find travelling to/accessing a food bank difficult so this is more flexible (8)
- Parents can be signposted to support at the same time (9)
- Parents might not meet criteria for a food bank referral but still need formula (10)

Q32 Can you think of any other benefits of a voucher approach?

________________________________________________________________

________________________________________________________________

________________________________________________________________

________________________________________________________________

________________________________________________________________

Q33 Can you see any risks to a voucher approach? Please select all that apply

- It restricts parental choice/autonomy (1)
- Receiving vouchers could feel stigmatising (3)
- It is more administratively complex than giving formula (4)
- The service / person might not be available at the right time (5)
- Parents might not want to admit to a health professional or local authority etc that they are struggling to feed their baby (6)
- Shops might accept vouchers for other things (7)
- It puts added pressure on health services / local authority / charities (8)
- It's more time consuming for parents who have already visited a food bank (10)

Q34 Are there any other risks you can see to a voucher approach?

________________________________________________________________

Q35 If you think about the potential options above, which do you think would work best?

- Access to a voucher from a health professional, charity or similar (3)
- Access via the foobank (5)

Q37 Please explain your answer above on preferences for access

________________________________________________________________

________________________________________________________________

________________________________________________________________

________________________________________________________________

________________________________________________________________

Q36 Thinking about the different options to access formula milk

|  | Strongy agree (1) | Agree (2) | Neither agree nor disagree (3) | Disagree (4) | Strongly disagree (5) |
| --- | --- | --- | --- | --- | --- |
| I think the best method can differ for different families (1) |  |  |  |  |  |
| We should have multiple access options available to make it easier for families (6) |  |  |  |  |  |
| I think the best method can differ in different situations / contexts (7) |  |  |  |  |  |
| Families should have a choice of method according to preference (8) |  |  |  |  |  |

Q68 Do you think the government should intervene and place a price cap/ limit on formula costs e.g. stop companies from making significant profits?

- Strongly agree (1)
- Agree (2)
- Neither agree nor disagree (3)
- Disagree (4)
- Strongly disagree (5)

Q69 Would you like to explain further about why you are in favour or not of price limits / caps for formula milk?

- Click to write Choice 1 (1)
- Click to write Choice 2 (2)
- Click to write Choice 3 (3)

Q70 Do you think the government should develop and offer a 'not for profit' national formula that is at cost price to families?

- Strongly agree (1)
- Agree (2)
- Neither agree nor disagree (3)
- Disagree (4)
- Strongly disagree (5)

Q71 Would you like to explain further about why you are in favour or not of a 'not for profit' milk?

________________________________________________________________

________________________________________________________________

________________________________________________________________

________________________________________________________________

________________________________________________________________

Q67 Some people have argued that one way to support parents would be allow shops to advertise offers on formula milk, or to allow loyalty points to be given for purchases or used to buy formula. Do you think shops should be able to

|  | Strongly agree (1) | Agree (2) | Neither agree nor disagree (3) | Disagree (4) | Strongly disagree (5) |
| --- | --- | --- | --- | --- | --- |
| Allow customers to spend loyalty points on formula (1) |  |  |  |  |  |
| Allow customers to get loyalty points on formula purchases (2) |  |  |  |  |  |
| Put offers on first stage formula milk (3) |  |  |  |  |  |

Q61 How much do you agree with the following statements?

|  | Strongly agree (1) | Agree (2) | Neither agree nor disagree (3) | Disagree (4) | Strongly disagree (5) |
| --- | --- | --- | --- | --- | --- |
| It would take a long time to accumulate enough points to make a difference (1) |  |  |  |  |  |
| It wouldn't make that much difference in the grand scheme of things as you can spend points on other items (4) |  |  |  |  |  |
| Not all the shops parents use have loyalty schemes (13) |  |  |  |  |  |
| Offers might not be available for preferred formula (5) |  |  |  |  |  |
| Offers might not be available when needed (6) |  |  |  |  |  |
| It would feel more equal i.e. You can buy / receive points for other items so why not formula (7) |  |  |  |  |  |
| It would help parents occasionally in an emergency e.g. as a back up (8) |  |  |  |  |  |
| It would feel less stigmatising (9) |  |  |  |  |  |
| A lower set price in the first place would feel more reliable (10) |  |  |  |  |  |
| Offers are about increasing sales not helping parents (11) |  |  |  |  |  |
| Offers might be used to encourage purchase of more expensive products when these are not necessary (12) |  |  |  |  |  |
| It would allow more competition between brands (14) |  |  |  |  |  |
| It would reduce reliance on one brand if parents moved between offers (15) |  |  |  |  |  |
| Companies don't use offers to reduce prices for the consumer - they use it to increase sales so parents wouldn't benefit in the long run (16) |  |  |  |  |  |

Q66 Do you have any other thoughts about shops being able to use offers / loyalty point schemes on formula in relation to supporting parents with cost?

________________________________________________________________

________________________________________________________________

________________________________________________________________

________________________________________________________________

________________________________________________________________

Q38 Thinking about the topic of feeding babies, how much do you agree with the following statements?

|  | Strongly agree (1) | Somewhat agree (2) | Neither agree nor disagree (3) | Somewhat disagree (4) | Strongly disagree (5) |
| --- | --- | --- | --- | --- | --- |
| Breastfeeding helps to protect the health of mothers and babies (1) |  |  |  |  |  |
| Formula being easily accessible can undermine breastfeeding (2) |  |  |  |  |  |
| We should encourage and promote breastfeeding (3) |  |  |  |  |  |
| The main thing is a baby is fed (4) |  |  |  |  |  |
| It doesn't really matter in the long run whether babies are breast or formula fed (5) |  |  |  |  |  |
| We mustn't risk making parents feel judged for how we feed their baby when we think about formula access (6) |  |  |  |  |  |
| Formula companies often take advantage of situations such as formula poverty to promote their products (7) |  |  |  |  |  |
| Formula companies want to do their best to support parents (8) |  |  |  |  |  |
| Parents feel a lot of judgement about feeding their baby so we should avoid talking about breastfeeding (9) |  |  |  |  |  |
| All first stage infant formulas available in shops meet a baby's nutritional needs regardless of brand or price (11) |  |  |  |  |  |

End of Block: Voucher schemes for purchasing infant formula

Start of Block: Other models

Q39 Other than food/baby bank distribution and voucher schemes, please tell us about any other models you are aware of that currently help food insecure parents obtain infant formula

________________________________________________________________

________________________________________________________________

________________________________________________________________

________________________________________________________________

________________________________________________________________

Q40 Please share if you have any thoughts about other potential ideas you have for models which could help food insecure parents obtain infant formula

________________________________________________________________

________________________________________________________________

________________________________________________________________

________________________________________________________________

________________________________________________________________

Q41 If you have any final thoughts, please share them here

________________________________________________________________

________________________________________________________________

________________________________________________________________

________________________________________________________________

________________________________________________________________

End of Block: Other models

Start of Block: Further involvement

This is the end of the survey, thank you for your time.  If you would like to help us further with our research, we are looking for stakeholders to be interviewed 1:1 online by a member of our research team to explore this issue in more depth.  We would also like to send you a copy of our study findings. Please select any / all that you'd like to be sent/ invited to.

- Study summary (4)
- Interview (5)

Q59 Please enter your email address below if you would like to receive a summary or potentially be contacted for an interview.

________________________________________________________________

________________________________________________________________

________________________________________________________________

________________________________________________________________

________________________________________________________________

Q55 If you need more resources to support parents:  Parents can find local 'Worrying About Money? resources to help identify local advice and support in your area Parents may be able to access support through their local authority Turn2Us has a cash grants search available for people facing financial problems StepChange offers free debt advice. They can search for their nearest IFAN food aid provider, their nearest Trussell food bank and their nearest Salvation Army venue. They may need a referral from an agency to access some food banks They can contact Mind for mental health support. The Samaritans also offers 24/7 emotional support for people struggling with their mental health - call 116 123 or email jo@samaritans.org If they are experiencing homelessness you could contact Crisis or Shelter for help Women's Aid offer information and support on domestic abuse If you need medical help contact NHS 111 online If you would like to read more about supporting families in financial crisis with formula milk you might find the following useful  https://www.unicef.org.uk/babyfriendly/wp-content/uploads/sites/2/2023/02/UNICEF-UK-Baby-Friendly-Guide-for-Local-Authorities-and-Health-Boards.pdf  https://www.unicef.org.uk/babyfriendly/wp-content/uploads/sites/2/2022/10/Infosheet-on-provision-of-infant-formula-for-families-experiencing-food-insecurity.pdf

End of Block: Further involvement
