# Supplementary material for: Infant Formula Affordability Negatively Impacts Parental Wellbeing, Financial Security and Safe Feeding Practices in the UK
Source: Matern Child Nutr. 2026 Jul 31;22(3):e70228. doi: 10.1111/mcn.70228 (PMC13425624; doi:10.1111/mcn.70228)
Supplement: Supplementary file 3 — Supporting File 3 [file MCN-22-e70228-s002.docx]

**Appendix three: Examples of interview questions for parents and stakeholders**

| **Parents** | **Stakeholders** |
| --- | --- |
| Which brand or type of formula are you currently using? What made you decide to use that one?  How easy or difficult is it for you to buy the formula your baby needs? How does this make you feel?  How often do you find it difficult to get the formula your baby needs?  Are there any other ways you’ve managed to get hold of formula other than buying it in a shop?  Have you ever had to cut corners or do things you thought might not want to because you couldn’t afford enough formula?  Do you think there is enough support for parents who are struggling to feed their baby? | We have heard in the media that lots more parents are struggling to afford formula milk for their baby. Do you feel that you’ve seen or heard of an increase?  What do you think, or hear the reasons are for parents struggling to buy formula milk?  How often do you personally come across or hear about parents who are struggling with the cost of infant formula? What impact is it having?  What impact do you see or think the high costs of infant formula are having for families? |
